# Precise Regulation of Gene Expression Dynamics Favors Complex Promoter Architectures

**DOI:** 10.1371/journal.pcbi.1000279

**Published:** 2009-01-30

**Authors:** Dirk Müller, Jörg Stelling

**Affiliations:** Department of Biosystems Science and Engineering and Swiss Institute of Bioinformatics, ETH Zurich, Basel, Switzerland; The Hebrew University, Israel

## Abstract

Promoters process signals through recruitment of transcription factors and RNA polymerase, and dynamic changes in promoter activity constitute a major noise source in gene expression. However, it is barely understood how complex promoter architectures determine key features of promoter dynamics. Here, we employ prototypical promoters of yeast ribosomal protein genes as well as simplified versions thereof to analyze the relations among promoter design, complexity, and function. These promoters combine the action of a general regulatory factor with that of specific transcription factors, a common motif of many eukaryotic promoters. By comprehensively analyzing stationary and dynamic promoter properties, this model-based approach enables us to pinpoint the structural characteristics underlying the observed behavior. Functional tradeoffs impose constraints on the promoter architecture of ribosomal protein genes. We find that a stable scaffold in the natural design results in low transcriptional noise and strong co-regulation of target genes in the presence of gene silencing. This configuration also exhibits superior shut-off properties, and it can serve as a tunable switch in living cells. Model validation with independent experimental data suggests that the models are sufficiently realistic. When combined, our results offer a mechanistic explanation for why specific factors are associated with low protein noise in vivo. Many of these findings hold for a broad range of model parameters and likely apply to other eukaryotic promoters of similar structure.

## Author Summary

Combinatorial regulation of gene expression is an important mechanism for signal integration in prokaryotes and eukaryotes. Typically, this regulation is established by transcription factors that bind to DNA or to other regulatory proteins. Modifications of the DNA structure provide another layer of control, for instance, in gene silencing. However, it is barely understood how complex promoter architectures determine key features of promoter dynamics such as gene expression levels and noise. Here, we employ realistic mathematical models for prototypical promoters of yeast ribosomal protein genes as well as simplified versions thereof to analyze the relations among promoter design, complexity, and function. By comprehensively analyzing stationary and dynamic promoter properties, we find that functional tradeoffs impose constraints on the promoter architecture. More specifically, a stable configuration in the natural design results in low transcriptional noise and strong co-regulation of target genes in the presence of gene silencing. Combined, our results offer a mechanistic explanation for why specific factors are associated with low protein noise in vivo. We expect that many of these findings apply to other promoters of similar structure.

## Introduction

Combinatorial regulation of gene expression is an important mechanism for signal integration in prokaryotes and eukaryotes (reviewed in [1]). Typically, specific motifs in the DNA sequence favor binding of particular transcription factors (TFs) and thus encode a *cis*-regulatory input function [2]. Protein-protein interactions among different TFs, which do not necessarily involve direct contacts with DNA, contribute to—frequently synergistic—regulatory function [1]. This is a very versatile mechanism for hierarchical control, e.g., when TFs can only be recruited in a pre-defined sequence or when they are excluded under specific conditions [3]. Chromatin state and chromatin-modifying activities provide yet another layer of regulation, and recruitment of the latter is typically also mediated by TFs [4]. Hence, multiple, complex levels of combinatorial control characterize transcriptional regulation [5].

New high-throughput measurement methods have generated a wealth of information on transcriptional regulatory circuits at different levels such as chromatin states, promoter occupancy by TFs, and mRNA expression dynamics as the system's output. Analysis of combinatorial regulation at the genome-scale points to a modular organization of transcriptional regulatory networks, which could facilitate data integration. However, this requires a multi-level analysis [6] and dynamic processes may lead to large functional re-arrangements of transcriptional regulatory networks. Concomitantly, understanding network design principles needs a detailed investigation of dynamic processes [7],[8].

Corresponding computational models aid in disentangling transcriptional network structures and in quantitatively analyzing the impact of promoter architecture on the regulatory outcome. Depending on network size, available experimental data, and model purpose, model types range from qualitative logical models to quantitative approaches based on thermodynamic considerations or ordinary differential equations (ODEs) (reviewed in [9]–[11]). However, most previous work focused on stationary gene–regulatory input functions in real-life organisms and in rational promoter design [2], [12]–[14]. Recently, stochastic kinetic models have received increased attention because we lack a deeper understanding of how gene network architecture shapes gene expression noise [15]. Stochasticity in gene expression arises from environmental effects and from intrinsic sources. It can have benefits and adverse effects for gene network function (reviewed in [16],[17]). Hence, noise in gene expression may be an evolvable trait that is intimately linked to promoter architecture [16]. For eukaryotic systems, irregular promoter activation due to chromatin modifications or transcriptional re-initiation are the main intrinsic noise sources [15],[18]. Despite recent progress [15],[18],[19], our understanding of how the dynamic interplay of transcription factors, chromosomal positioning, epigenetic control, and *cis*-regulatory promoter elements shapes expression dynamics and noise properties is still limited. Moreover, much of our knowledge derives from artificial expression systems and unnatural stimuli, and we need more studies in complex natural systems to reliably assess the impact of stochasticity on diseases and developmental pathways [16]. For this, integrated approaches have to consider potential tradeoffs between optimal noise properties, phenotypic fitness, high mRNA productivity, and robustness to perturbations [15],[20].

Budding yeast ribosome biogenesis can be employed for such an integrated analysis because the system is quantitatively well-characterized, complex, and crucial for cell physiology. It needs to operate efficiently and reliably; for instance, ribosome biogenesis requires coordinated expression of several hundred genes and accounts for up to 80% of transcriptional activity during rapid growth [21],[22]. Tight and coordinated transcriptional control therefore appears critical, and promoter architecture plays a key role in integrating TF interactions and different layers of control. The system employs the Forkhead (FH)-type TF Fhl1 (accession number S000006308; all accession IDs refer to the Saccharomyces Genome Database (SGD) available at http://yeastgenome.org unless mentioned otherwise), which belongs to a family of transcriptional regulators with more than 100 members conserved from yeast to human. These regulators typically serve as converging points for signaling pathways, they possess variable activation/repression domains, and often act in concert with coactivators/corepressors and general regulatory factors (GRFs) (for reviews cf. [23],[24]). Moreover, binding of the GRF Rap1 (SGD S000005160) and of Fhl1 in yeast highly correlates with low protein noise [25] and transcriptional co-regulation is particularly strong for RP genes [26],[27], but the causes for both are unclear. These features make the control of ribosome biogenesis an ideal example system to address three general questions: Do complex promoters provide advantages over alternative, simpler designs? Why are complex designs frequently employed when reliable regulation is critical? Is the complex promoter architecture especially suited to provide low variations in mRNA levels?

Here, we address these questions by developing, analyzing, and validating a set of dynamic mathematical models of the promoter of yeast ribosomal protein (RP) genes. The set includes the in vivo design and three functionally related, but progressively simpler synthetic architectures. We integrate selected information from large-scale studies and from targeted experiments to provide the necessary quantitative basis for these models, and to comprehensively characterize the stationary and dynamic regulation of promoter activities using deterministic and stochastic simulations. This enables us to pinpoint structural features underlying the observed behavior and to identify functional tradeoffs that impose constraints on promoter architecture.

## Results

### A Set of Promoter Models

**Figure 1:**
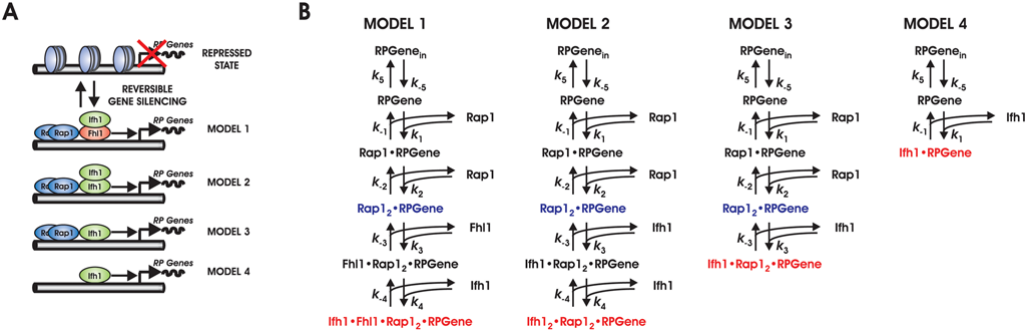
Architecture of ribosomal protein gene promoters and corresponding models. (A) Alternative promoter structures considered for wild-type architecture (Model 1) and for progressively simplified synthetic configurations (Models 2–4). (B) Reaction networks of the individual models describing progressive association of TFs until full activation. Promoter states with basal and full transcriptional activity are marked in blue and red, respectively.

To develop kinetic promoter models, we start from elementary interactions between transcription factors and DNA. Typical RP gene promoters contain paired binding sites for the GRF Rap1 [27] (cf. Figure 1A and 1B). Rap1 binds DNA directly [28], which is required for efficient expression of RP genes and maintains promoter regions essentially nucleosome-free [3],[29]. This complex alone can recruit RNA polymerase II for basal transcriptional activity [27],[30]. Recruitment of the FH-type transcription factor Fhl1, which in turn binds Ifh1 (SGD S000004213) via its Forkhead-associated (FHA) domain, leads to full activation [27],[31],[32]. When Fhl1 is bound in the absence of Ifh1, even basal transcription is suppressed [33]. Upstream signaling pathways can convey nutrient status to Ifh1 such that it rapidly dissociates from the promoter. This leads to a substantial reduction of RP transcription, whereas occupation by Rap1 and Fhl1 remains unchanged [27],[32]. High-confidence datasets show that this RP gene promoter architecture is very generic in yeast [31],[32]. Although additional regulators can contribute to RP gene regulation, their effects are probably indirect, strain-specific, or they affect RP gene expression in the same qualitative fashion as Ifh1 [27],[29],[34],[35]. Hence, the interactions between GRF, Fhl1, Ifh1, and RP gene promoters capture the key aspects of transcriptional control of RP genes.

Model 1 represents the wild-type scenario as follows (Figure 1B): Sequential recruitment of two Rap1 molecules leads to basal transcriptional activity. Subsequent Fhl1 binding in the absence of Ifh1 quenches basal transcription, while Fhl1-dependent recruitment of Ifh1 induces full activity. Since regulation of Ifh1 binding to the promoter critically determines promoter activity [31],[32] we simulate regulation upstream of Ifh1 by varying the amount available for promoter binding, i.e., the effective Ifh1 concentration (see Protocol S1 for details).

Since most physiological stimuli appear to regulate Ifh1 binding while Rap1 and Fhl1 serve as scaffold, it is unclear if the seemingly complex architecture of the natural RP gene promoter yields any functional advantage. In principle, one could envision the same coordinated regulation by controlling the activity, localization, or DNA binding affinity of a *single* TF such as Ifh1. Note that more complex promoters in terms of combinatorial control exist even in yeast [36]. However, RP genes are special because they form an exceptionally tight cluster of coregulated genes in transcriptome studies [26],[27].

To investigate differences in function and regulatory performance of structurally related, but simpler architectures, we developed three alternative promoter models (Models 2–4). They are progressive simplifications of the natural promoter configuration (Figure 1B; see Protocol S1 for details). Models 2 and 3 follow the same logic of sequential TF recruitment as the wild-type model. In Model 2, a second Ifh1 molecule replaces Fhl1 and transcription ceases when only a single Ifh1 is bound (Figure 1B). By contrast, recruiting one Ifh1 molecule suffices for full activation in Model 3 and Model 4. Compared to the wild type (Model 1), Model 2 is a biologically more parsimonious solution with only two different proteins, but it maintains the same kinetic order as Model 1. Model 3, in addition, has a reduced kinetic order. Finally, Model 4 is the structurally simplest promoter variant that can transmit environmental inputs to a target gene. Notably, it does not employ GRFs.

Although the simplified models are synthetic, they correspond to promoter architectures encountered in vivo. Model 2 with its cooperative activation by homodimeric TFs resembles regulation by cI repressor in phage λ [37]. Certain Ternary Complex Factor-type promoters are structurally similar to Model 3 [38]. Promoter architectures with single TFs as in Model 4 are well-described in yeast, e.g., involving the TF Gcn4 (SGD S000000735) in amino acid biosynthesis [36]. Importantly, for the molecular species denoted as Ifh1 in simplified Models 2–4 we assume *functional* equivalence, but not *structural* identity to Ifh1, which itself cannot bind to DNA [29].

Stochastic binding and dissociation events of TFs and of RNA polymerase determine whether a given RP gene is transcribed. We represented control events by sets of elementary chemical reactions and mass-action kinetics [39],[40] with or without including gene silencing due to changes in chromatin structures (see Material and Methods and below). In this modeling framework, derivation of promoter kinetics for both the deterministic regime (based on ODEs) and for the stochastic setting is straightforward [41]. To analyze mean promoter activities, or other average properties, we used a deterministic description and verified its qualitative consistency with stochastic simulations for selected models and parameter settings (data not shown). Some simulations were performed without considering gene silencing, both to separate its effects from those of the promoter configuration alone and because an equivalent stationary behavior could have been achieved in its presence by adapting the binding constants (see Materials and Methods and Protocol S1 for details).

### The RP Gene Promoter Encodes a Tunable Switch

**Figure 2:**
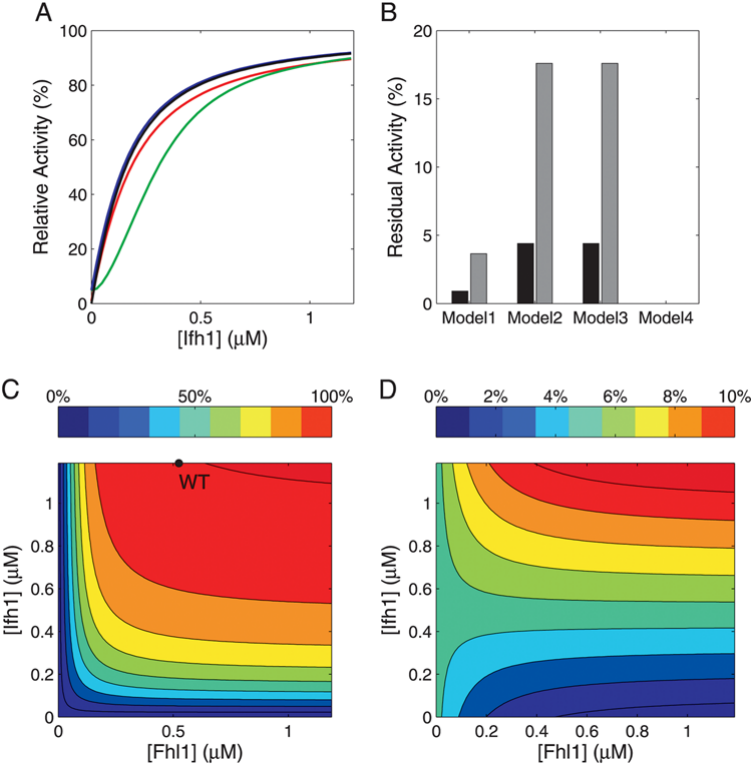
Stationary regulatory properties of the promoter designs. (A) Stationary input-output characteristics with variable Ifh1 input concentration (basal activity of *η* = 0.05) for models 1 (red), 2 (green), 3 (blue), and 4 (black). (B) Normalized residual promoter activity in the absence of Ifh1 for basal activities of *η* = 0.05 (black bars) or *η* = 0.20 (grey bars). (C and D): Promoter activity as a function of total Ifh1 and Fhl1 concentrations (*η* = 0.20) for wild-type model 1 (C) and for a variant with 100-fold decreased Ifh1 affinity (D). Color codes represent percent of maximum promoter activity and the black circle in (C) indicates the operating point in vivo.

To address how upstream signaling pathways—through variation in Ifh1 levels—modulate RP gene transcription, and how this is influenced by the ambivalent coactivator/repressor Fhl1, we compared model predictions of stationary promoter activity without chromatin remodeling. For realistic parameter values, promoter activities are very similar for all models (Figure 2A) because, in the more complex models 1–3, most genes are occupied by Rap1 dimers and thus available for Ifh1 binding. Notably, the in vivo configuration (Model 1) does neither provide the highest stationary activity, nor the steepest or the most graded response of all model variants. Hence, the stationary input-output characteristics with respect to Ifh1 alone do not explain the complexity of the in vivo architecture.

Next, we focused on gene inactivation because rapid down-regulation of ribosome synthesis is important for cellular growth when nutrients become scarce. In this case, Ifh1 leaves the promoter and RP synthesis effectively ceases, whereas environmental conditions barely affect Fhl1 and Rap1 binding [3],[42],[43]. We emulated adverse environmental conditions by complete absence of Ifh1. Only the simplest model without GRF (design 4) enables a complete shut-off (Figure 2B). All other configurations retain a basal activity due to RP gene complexes with two Rap1 molecules. For realistic values of basal promoter activity (*η*), Fhl1 binds the majority of Rap1_2_-RP gene complexes in design 1 and thereby efficiently quenches basal transcriptional activity when Ifh1 is absent (Figure 2C and 2D). In models 2 and 3, transcription could only be lowered by an inefficient 5–10-fold reduction of cellular Rap1 levels. While the qualitative model behavior results from the way Fhl1 and basal activation by Rap1 are represented, we need such realistically parametrized mathematical models to assess these control effects quantitatively. Thus, we suggest that the ambivalent coactivator/corepressor (Fhl1) enables a rapid switch between full and low basal activity without invoking inefficient control by GRFs. This may apply to similar promoters with dual coactivator/repressor TFs constitutively bound GRFs other than Rap1 [24],[38].

The analysis of model 4 demonstrates that a single-input promoter with efficient shut-off can be realized with a single transcription factor and without basal activity conferred by the GRF. We therefore analyzed the combined effects of Fhl1 and Ifh1 on promoter activity. By varying the Fhl1 concentration it is not only possible to adjust the degree of activation in the presence of Ifh1 and the degree of repression in its absence, but also the factor fold-change between the two states (Figure 2C). In other words, independent regulation of Fhl1 and Ifh1 provides an ON–OFF switch with basal activity and tunable upper and lower activity bounds. Predicting this behavior requires quantitative knowledge on protein levels and kinetic constants since, for example, decreasing the affinity of Ifh1 by 100-fold renders Fhl1 predominantly a repressor at low Ifh1 levels (Figure 2D). Promoter activity is sensitive to changes in Ifh1 over a wider concentration range compared to Fhl1; especially at wild-type Fhl1 levels, Ifh1 can robustly modulate the activity plateau (Figure 2C). By contrast, Fhl1 determines sensitivity of promoter activity to Ifh1: low effective Fhl1 concentrations limit the maximum promoter activity and make the promoter unresponsive to Ifh1 changes. Hence, both Fhl1 and Ifh1 can serve as input signals for tuning the switch.

These generic predictions are supported by experimental evidence that Fhl1 and Ifh1 respond to different regulatory inputs [36],[44],[45]. In addition, the models predict that effective regulator concentrations need to be considerably lower than total in vivo protein levels to establish a tunable switch (Figure 2A and 2C). This agrees with reports of large changes in nuclear Ifh1 and Fhl1 concentrations [34] and with estimates that much of Ifh1 is unavailable for promoter binding in vivo ([45], J. Merwin and D. Shore, personal communication). Full exploitation of the complex promoter architecture's regulatory potential, hence, requires regulatory mechanisms that target both inputs individually. This suggests novel regulatory motifs in the control of yeast ribosome biogenesis.

### Qualitative Model Behaviors Are Robust

Model predictions may depend on the choice of binding affinities between TFs and DNA as well as between the TFs themselves. Naturally, the question arises to what extent the relative model performance can be generalized. Optimizing each model's parameters separately over a broad parameter range demonstrates that the relative performance of promoter variants regarding maximum activity and shut-off properties remains unchanged (cf. Figure S5 and Protocol S1 for details). However, the use of such ‘optimal’ parameter sets can be problematic because the evolutionarily relevant objective function is unknown. As a complementary approach, we employed robustness analysis based on the natural promoter structure and choice of TFs because they represent the known outcomes of evolution. More specifically, we quantified the robustness of model predictions by assaying the sensitivity of achievable promoter activity to random perturbations in TF binding constants (see Materials and Methods). This informs us to what extent a specific prediction depends on a particular choice of system parameters. Insensitivity to parameter variations justifies generalizations, especially because robustness to random perturbations is an important characteristic of functional biological networks [46],[47].

**Figure 3:**
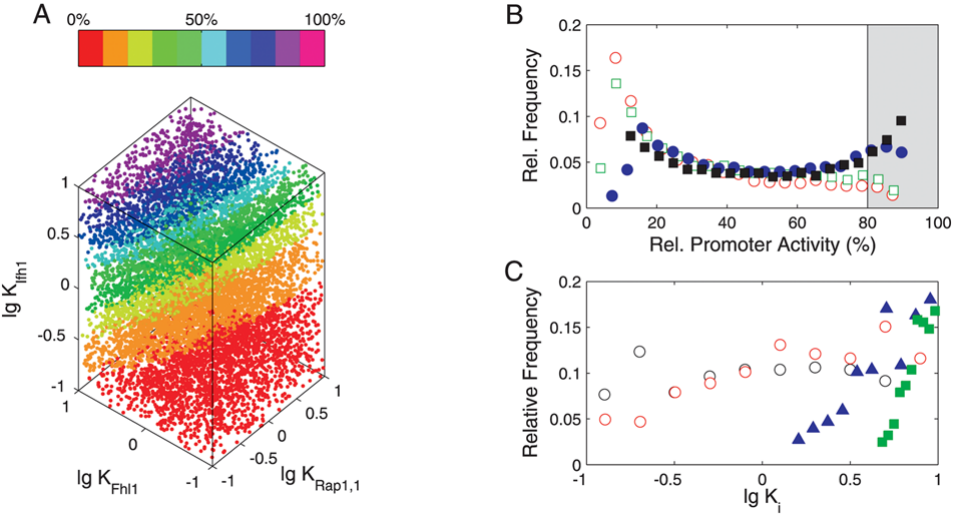
Robustness of promoter activity against random perturbations in binding constants. (A) Color-coded relative stationary promoter activity for *n* = 10,000 simulations of log-uniformly, randomly sampled parameter combinations. (B) Distribution of stationary promoter activities for 10,000 vectorially-perturbed parameter sets per model (red – Model 1, green – Model 2, black – Model 3, blue – Model 4). Parameters were varied according to a log-uniform distribution. (C): Affinity combinations yielding high promoter activity in the natural design (Model 1): distributions of those *n* = 405 parameter combinations out of 10,000 samples that resulted in >80% of maximal activity. Black – *K_Rap1,1_*, red – *K_Rap1,2_*, blue – *K_Fhl1_*, green – *K_Ifh1_*. All equilibrium constants *K* are in µM^−1^.

Figure 3A shows the promoter activity for Model 1 as a function of binding affinities for Rap1 (1st step), Fhl1, and Ifh1. The pronounced vertical stratification demonstrates that strong Ifh1 binding is essential for high promoter activity. The affinity of Fhl1 has a less marked effect and the attainable promoter activity barely depends on the strength of the first and second Rap1 binding steps (Figure 3C, Figure S1, and Figure S2). These features apply to all models (data not shown) and they are, thus, rather independent of the actual promoter configuration. The distribution of binding affinities associated with high promoter activity in Model 1 confirms that early binding steps are less sensitive to changes in binding affinities than later ones (Figure 3C). Maximum sensitivity in a sequence of cooperative binding steps is known to require high association constants immediately before RNA polymerase binds [13],[37]. Our analysis generalizes this result to promoters that have intermediate states with basal activity when realistic concentrations and binding constants are considered.

However, the more complex designs were less robust when we mutated all binding affinities simultaneously. Only 4% of the mutated promoters showed high stationary activity for Model 1, as opposed to 6% for Model 2, 17% for Model 3, and 22% for the simplest Model 4 (Figure 3B). Ifh1 and Rap1 should contribute similarly to sensitivity and insensitivity in designs 1–3. The major difference in robustness must, hence, be conferred by the additional intermediate state without transcriptional activity in models 1 and 2 (see Figure 1, e.g., transcriptionally inactive, single-bound Ifh1 in model 2). Apparently, the decreased robustness in Model 1 is the trade-off for added functionality. The ubiquitous presence of FH-type regulation and its usage at critical control points suggest that additional flexibility outweighs potential effects of reduced robustness. This holds for a broad, physiologically plausible range of transcription factor affinities. It will, therefore, generalize to other structurally related promoters that employ combined coactivator/corepressor TFs.

### Promoter Architectures Differ in Resistance to Chromatin Remodeling

Efficient regulation crucially depends on the ability to consistently respond to changes in the input(s). Next, we therefore investigated the dynamic promoter responses to varying external conditions. More specifically, we investigated the dynamic promoter performance with and without gene inactivation due to chromatin modifications, which may lower the concentration of accessible genes at any given time point. For the steady-state analysis above, silencing could be mimicked by decreasing the affinity of TFs for DNA, but this does not hold for the dynamic behavior.

Specifically, we mimicked environmental changes by applying a sinusoidal time-varying input of free Ifh1 with fixed amplitude and frequency. Such a periodic forcing function is the standard choice in frequency response analysis [48] because the system is stimulated by a single frequency, and not by a frequency spectrum as for other input shapes. This allows us to map output behavior to a unique input frequency. For linear models, input and output frequencies match and only a phase shift occurs, while nonlinear models produce a spectrum of output frequencies. By varying the input frequency, we can mimic noise effects (high frequencies) and observe how well the system tracks dynamic inputs (lower frequencies).

**Figure 4:**
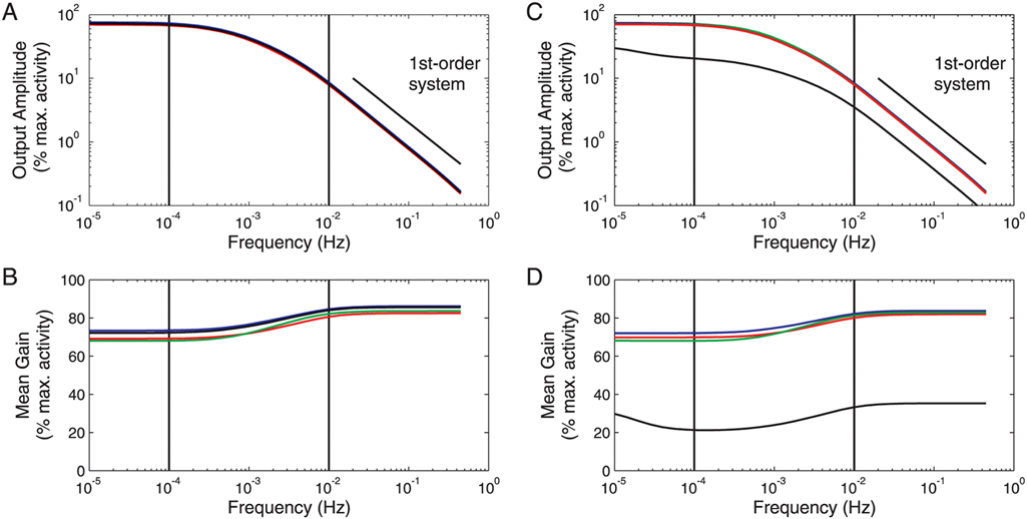
Frequency response of promoter architectures. Bode-type plot comparing model responses to a sinusoidal input in the concentration of free Ifh1 in the absence (A,B) or presence (C,D) of gene inactivation. (A) and (C): normalized amplitude of promoter activity oscillations; (B) and (D) average promoter activity. Color codes: Model 1 – red, Model 2 – green, Model 3 – blue, Model 4 – black. Solid vertical markers roughly delineate the physiologically relevant frequency range (*f* = 10^−2^–10^−4^ Hz corresponding to periods between ∼1.7 min and ∼2.8 hours).

Figure 4A shows how the scaled amplitude of promoter activity oscillations and the frequency-dependent average promoter activity (Figure 4B) vary for oscillation periods between 1 s (*f* = 1 Hz) and approximately 27 hours (*f* = 10^−5^ Hz) when gene silencing is neglected. Along with the phase shift between input and output activity, this representation is related to the well-known Bode plot for linear systems in control theory. It combines the ratio of output and input amplitudes in a double logarithmic plot and the phase shift between output and input in a semi-logarithmic plot as function of the input frequency. As our models are nonlinear, the predicted promoter activities deviate from the sinusoidal shape of the input and show a more switch-like behavior, but the predominant frequency contribution to the output was always identical to the input frequency (see Figure S3). Shape modulation causes differences between the average promoter activities for dynamic and constant inputs, namely lower/higher activities for slow/fast Ifh1 oscillations, respectively (Figure 4B). In all designs, promoter activity follows slow input signals quantitatively and closely for periods larger than 15 minutes (*f*<10^−3^ Hz), while it rejects fast Ifh1 oscillations with a period below 15 min (*f*>10^−3^ Hz) for the chosen parameter settings. The frequency response of activity oscillations and the phase shift (Figure S4A) are characteristic of a first-order-type low-pass behavior, which enables faithful transmission of low-frequency signals and rejection of high-frequency noise in engineered and biological systems [11],[48],[49].

To analyze the impact of random chromatin modifications on promoter dynamics, we assumed a reversible and constitutive process that maintains a compact chromatin state (assembled nucleosomes) in the absence of TF binding (see Protocol S1). With a single TF (Model 4), dynamic gene inactivation substantially decreases the average promoter activity, alters the phase response, and suppresses activity fluctuations (cf. Figure 4C and 4D and Figure S4C and S4D). The latter leads to a desirable noise filtering at high frequencies, but it also prevents faithful input tracking in the physiologically relevant frequency range. A stronger TF binding affinity can compensate for the low average activity (data not shown). However, faster association rates may meet physico-chemical limitations [50], while slower dissociation will increase the response time to input signals.

In contrast, chromatin closure has almost no effect on the dynamics of Rap1-containing promoter architectures (Models 1–3), apart from a slight reduction in average promoter activity. Similarly, promoter activity in models 1–3 resists noise even for large, physiologically plausible fluctuations in Rap1 concentrations regardless of chromatin compaction (Figure S4B and S4D and data not shown). Importantly, this superiority of Rap1-containing architectures is not restricted to a specific choice of parameters—and, hence, TF-binding site affinities—nor to a specific stimulus shape: We obtained the same qualitative behavior when optimizing the parameters of each model separately for the response to step changes in Ifh1 within a range of realistic kinetic constants and TF affinities (Figure S5 and data not shown; see Protocol S1 for details). Moreover, optimal parameter sets obtained in independent optimization runs showed parameter variability in agreement with the above robustness analysis (see Protocol S1).

Altogether, stably bound dimeric GRFs, in general, can protect the promoter from noise propagation due to unspecific chromatin modifications. GRFs ensure that the promoter remains in a *poised* state for rapid reactivation even after prolonged absence of TF binding. This obviates the need to first reactivate the genes in a sequence of—potentially slow—chromatin modification steps [15],[16],[18] before the transcriptional machinery can be recruited again. This interpretation is in line with the observation that Rap1 maintains RP gene promoters essentially nucleosome-free [3],[29] and that it is necessary and sufficient for TFIID recruitment [51]. It is also consistent with the proposed barrier function of Rap1 in preventing spreading of silent chromatin [28]. Hence, we propose that the natural RP gene promoter architecture ensures efficient promoter activation and rapid responses to input signals even when unspecific chromatin modifications occur.

### Reduced Noise Transmission of Rap1-Containing Promoters

**Figure 5:**
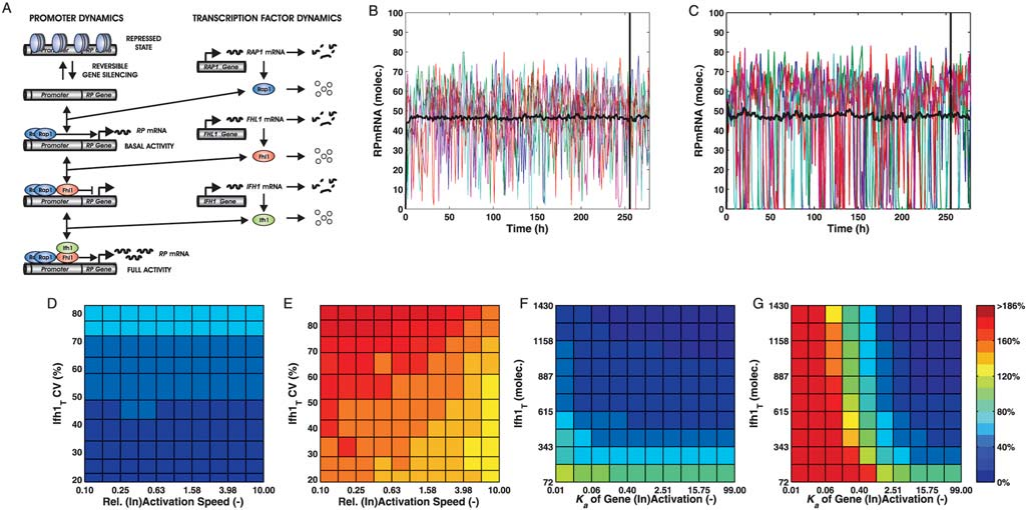
Rap1-containing promoters exhibit superior noise suppression. (A) Example of an extended stochastic model including TF noise based on Model 1. (B–G) Noise levels (CV) of RPmRNAs with gene silencing and noisy transcription factor levels as determined by stochastic simulation for Model 1 (B,D,F) and Model 4 (C,E,G). For each parameter combination, 500 simulations into steady state were performed. (B) and (C): Representative simulation time courses and mean trajectory (*n* = 500) of RPmRNA dynamics at intermediate silencing strength (*K_a_* = 0.40) for Model 1 (B) and Model 4 (C). Black vertical marker: starting point of sampling for CV evaluations. For a fair comparison, Ifh1 levels yielding equivalent mean RPmRNA levels are shown. (D) and (E): RPmRNA noise when simultaneously varying the speed of gene silencing (relative to the nominal value) and total Ifh1 levels (for *CV_Ifh1_T_*≈*const*); (F) and (G): RPmRNA noise as a function of the equilibrium constant of gene reactivation (*K_a_*) and of Ifh1 noise levels (*CV_Ifh1_T_*) for a constant Ifh1 level (430 molecules).

The deterministic analysis suggested that Rap1-containing promoters are more resistant to noise from random chromatin modifications. To further investigate noise propagation, we analyzed the ‘extreme’ models 1 and 4 by stochastic simulations. In addition to chromatin modification, we considered inherent fluctuations of TF levels as noise sources (see Figure 5A and Materials and Methods). A priori, it is therefore not obvious if the architecture with a single TF or the more complex design with three noisy TFs transmits more noise to downstream mRNA production.

In particular, we focused on the stationary noise in RP mRNA levels as a function of four factors (see Figure 1 for the corresponding reactions): (i) the level of Ifh1 as the main dynamic TF, assuming a constant coefficient of variation (CV) for this TF, (ii) the noise associated with a constant Ifh1 level, (iii) the equilibrium constant of chromatin compaction for a constant inactivation rate, and (iv) the velocity of compaction for a fixed equilibrium constant. Figure 5B and 5C show example simulations for models 1 and 4, respectively, where model parameters are adjusted such that both models generate equivalent average mRNA numbers for the same compaction efficiency. Here, mRNA levels in the simple model drop to very low values much more frequently than for the GRF-containing design, causing increased variation in mRNA numbers (see also Figure S6). This is a first confirmation of the predictions on noise resistance from the deterministic analysis.

To investigate gene expression noise more systematically, we explored the combined effects of variations for pairs of the above influence factors. Noise was quantified by calculating the CV of mRNA numbers for simulated trajectories in steady-state. In the presence of chromatin remodeling, the natural promoter architecture (model 1) exhibits lower mRNA noise than the simple design in all conditions investigated (Figure 5D–G). Notably, mRNA level variations in design 1 are essentially independent of either the velocity (Figure 5D) or the strength (Figure 5F) of compaction. Increasing Ifh1 noise levels only has a moderate effect in Model 1 and mRNA noise responds more to changes in Ifh1 numbers. By contrast, the simple architecture 4 is inherently more sensitive to the influence of Ifh1 noise levels and chromatin remodeling, especially if compaction is efficient or the chromatin opening/closing cycle is slow (Figure 5E and 5G). The two designs display similar low mRNA noise levels, or even better performance of Model 4, only when compaction is inefficient (*K_a_*≥2.51, i.e. when RP genes are active more than 70% of the time in the absence of any TFs). We conclude that, despite its complexity, the natural design specifically prevents random fluctuations caused by chromatin remodeling.

**Figure 6:**
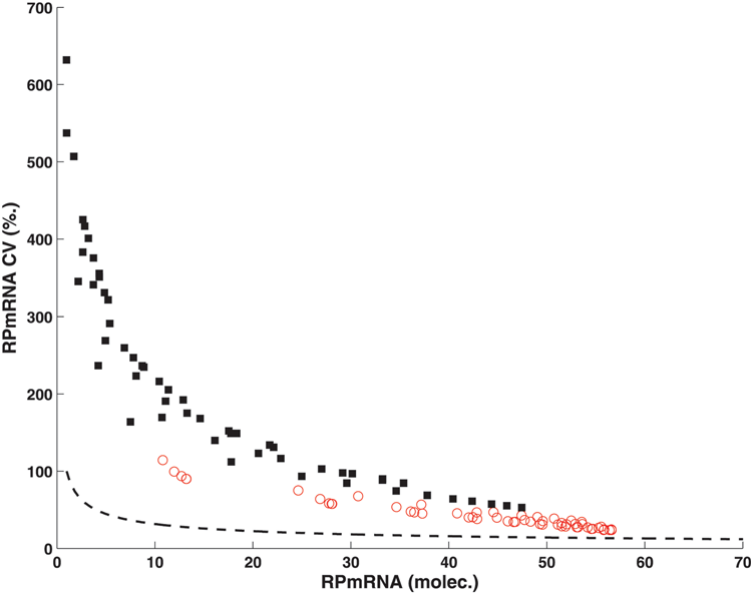
Dependence of noise levels on mRNA numbers. Noise levels of RPmRNAs as a function of mean mRNA numbers for *K_a_* values below 0.4 and the full range of Ifh1 levels analyzed. Open symbols: Model 1, filled symbols: Model 4. The dashed line indicates the expected Poissonian distribution due to random fluctuations in mRNA numbers without promoter influences.

What are the sources for the lower noise in gene expression of the natural design? Apparently, scenario 4 produces fewer mRNA molecules than scenario 1 because constitutive chromatin compaction inactivates a higher fraction of promoters. However, a systematic comparison of relative variability for a range of mRNA levels demonstrates that promoter configuration 1 is consistently associated with less noise than design 4 (Figure 6). The majority of mRNA variation—especially for low mRNA levels—results from irregular promoter activation as indicated by comparison with the expected noise levels due to discrete mRNA numbers (leading to Poissonian fluctuations) alone. Noise reduction for the natural architecture only minimally depends on the basal activity of the Rap1_2_-RP gene complex (not shown)—it almost exclusively results from the promoter structure.

Hence, noise creation and propagation at complex promoters is not solely determined by the binding strength of a particular TF, but also critically depends on the order of recruitment and on dynamic interactions with other TFs. Consequently, the domains mediating protein–protein interactions among cooperating TFs are selectable targets for the evolution of noise traits.

More specifically, Rap1-containing promoters achieve low intrinsic noise in gene expression because they minimize stochastic noise induced by unspecific remodeling events, especially for realistic kinetic values and molecule numbers in yeast. Importantly, the simulation results in Figure 5 and Figure 6 demonstrate that this model prediction is robust even when key parameters are perturbed several fold from their nominal values.

The regulation of RP genes is intricate because transcriptional co-regulation is particularly strong [26],[27]. Co-regulated promoter activation is key to induce and maintain concerted expression of gene sets that are required simultaneously. To quantify the degree of mRNA coexpression from individual but identical RP gene promoters, we used the sum of squared pairwise differences between mRNA molecule numbers over time (cf. Protocol S1 for details). The average sum is much smaller for Model 1 than for Model 4 and the differences between natural and simple design are highly significant (p<10^−36^, Welch's *t*-test). Hence, the natural promoter architecture is clearly superior in keeping absolute mRNA levels within tight bounds for large gene sets simultaneously. It enables efficient production of molecular machine precursors in stoichiometric quantities despite short mRNA half-lives that are required for quick adaptation to changes in external conditions. For ribosomal proteins, these features are essential because, when environmental conditions deteriorate, resource-intensive ribosome synthesis must be stopped immediately to reroute building blocks and energy to processes critical for survival.

### Model Validation

Finally, to critically test the predictive capabilities of the most realistic model (Model 1) we used two independent data sets for model validation. In both cases, except for experiment-specific settings, model structures and parameters remained unchanged. More specifically, we compared model predictions with the experimentally observed dynamic response to *IFH1* overexpression to evaluate if model structure and parameters would yield reliable predictions for a regulator contained in the model.

**Figure 7:**
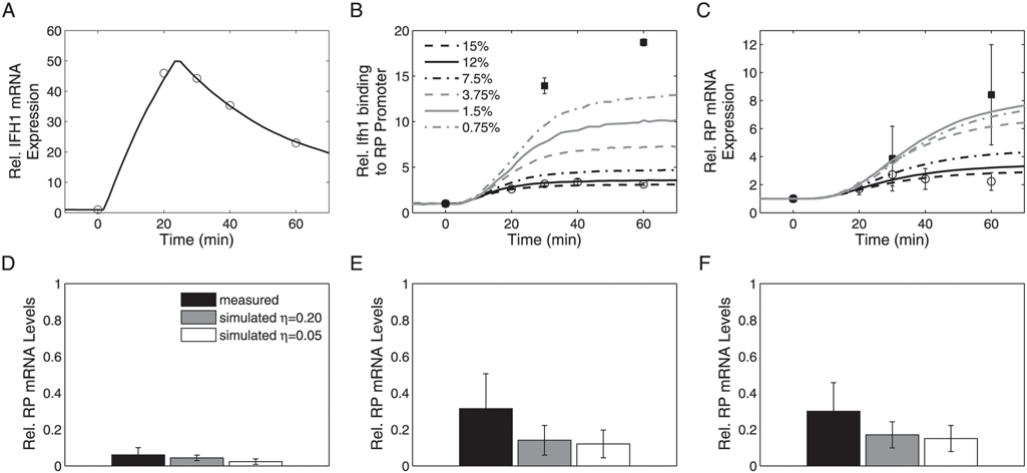
Validation of the most realistic model (Model 1). (A–C) Dynamic responses to induction of *GAL1*-controlled *IFH1* expression upon shifting from a non-inducing carbon source to galactose. Experimental data are shown as symbols (circles: data from Schawalder et al. [31] for glycerol+lactate→galactose, squares: data from Wade et al. [32] for raffinose→galactose). Model predictions (lines) are averages of *n* = 10,000 or 50,000 stochastic runs (*η* = 0.05). *IFH1* mRNA (A), Ifh1 promoter occupancy (B), and RP mRNA (C) for the fitted *IFH1* mRNA time course and varying basal *IFH1* expression (0.75–15% of WT level on glucose). In (B) and (C), error bars represent standard deviations of the mean based on *n* measurements; (B) open circles (*n* = 3), filled squares (*n* = 4), (C) open circles (*n* = 113), filled squares (*n* = 5). See Protocol S1 for details. (D–F) Predicted stress response of RP mRNA levels. Comparison of simulated and measured RP mRNA levels under various stress conditions (experimental data from [32]) for heat shock (D), osmotic shock (E), and rapamycin addition (F). Error bars indicate the standard deviation of the mean for three RP genes (*RPL2B*, *RPL27B*, and *RPS11B*; SGD S000001280, S000002879, and S000000252). Simulations were performed for basal promoter activities of *η* = 0.05 and *η* = 0.20, respectively. See Protocol S1 for details.

First, we compared the predicted dynamics of Ifh1 binding to RP promoters and RP mRNA production for galactose-inducible *IFH1* expression with experimental data [31],[32] (see Protocol S1 for details). Using a fit to the measured *IFH1* mRNA profile [31] as input (Figure 7A), Model 1 predicted promoter dynamics and RP mRNA production after induction of the *GAL1*-*IFH1* construct (*GAL1*: SGD S000000224). Since the absolute level of basal *GAL1*-*IFH1* expression under non-inducing conditions was not determined, we performed simulations for a range of plausible values. For basal *IFH1* mRNA expression at 12% of the wild-type level on glucose we obtained good qualitative and quantitative agreement between model and experimental data (Figure 7B and 7C), independently of the assumed basal activity *η* (data not shown). The model does not capture the decrease in measured mRNA levels at the last time point. However, no such reduction was observed in a similar experiment [32] (cf. filled squares in Figure 7C). We cannot exclude that deviations between model and data reflect, at least in part, unmodeled mechanisms. Interestingly, the model predicts that larger changes in Ifh1 occupancy at the promoter are not necessarily linked to a monotonic increase in the fold-change of RPmRNA levels (Figure 7B and 7C and Figure S7). Quantitative discrimination of model alternatives for this experimental setup, therefore, critically depends on accurate quantification of induction dynamics and *IFH1* mRNA basal levels in absolute terms; more comprehensive experiments are required to evaluate model performance more stringently.

To assess potential structural model uncertainties, we simulated the relation between Ifh1 promoter occupancy (which is the key control variable in the model) and RP mRNA production for stresses that might involve unmodeled regulators. In the model, stationary RP mRNA levels depend linearly on the fraction of Ifh1-bound RP promoters. This assumption leads to qualitatively correct predictions of changes in RP mRNA levels (Figure 7D–F) in response to heat shock, osmotic shock, and rapamycin addition [32]. Even quantitatively, the differences between measured and simulated responses were not statistically significant (Welch's *t*-test, 95% confidence level) in any of the three conditions. We obtained the same results for predicted Rap1 occupancies and for Fhl1 occupancy under osmotic shock (Figure S8). The difference between simulated and measured Fhl1 occupancies, however, was significant for heat shock (*p* = 0.0055) and rapamycin addition (*p* = 0.0084). Underestimation of Fhl1 binding in these conditions may reflect the influence of additional regulators. Such quantitative discrepancies highlight which model aspects require improvement; they identify possible settings under which alternative regulators can be studied in future experiments. We conclude that Model 1, which considers only Ifh1 and Fhl1 as dominating dynamic regulators, correctly captures salient features of RP gene promoter and mRNA behavior under stationary and dynamic conditions. Biologically meaningful and partly quantitative predictions are, hence, possible already with our simple model.

## Discussion

Complex promoters are involved in many cellular processes where correct timing of expression or precise and coherent regulation of gene sets is required. Their architectures, however, prevent intuitive explanations of promoter functions and advantages for controlling gene expression dynamics. Using the well-characterized yeast RP gene promoter as example, we derive a set of quantitative kinetic models for the natural and for three simplified synthetic promoter configurations. Our model comparison encompasses a broad range of performance characteristics, including dynamic responsiveness and noise transmission, which are not commonly covered in more traditional promoter models [9],[10].

For the specific example of yeast RP gene promoters, we conclude that the natural design is particularly suited to combine tunable regulation of gene expression with a fast response to external signals, strong co-regulation of target genes, and low mRNA noise in the presence of chromatin remodeling. These are partially contradicting objectives, and a quantitative analysis is required to evaluate the corresponding trade-offs. In particular, the natural promoter can serve as switch between activating and repressing modes with tunable upper and lower activity bounds. Despite the limited quantitative data available for model development, the most realistic models' qualitative—and to a certain extent quantitative—features and predictions comply with our knowledge on RP gene regulation in yeast. Importantly, several predictions are new and experimentally testable: (i) the importance of Forkhead proteins for superior shut-off properties and (ii) the differential regulation of Fhl1 and Ifh1 required for tunable switch function. Specifically, the role of Rap1-Fhl1 scaffolds in achieving low transcriptional noise mechanistically explain why RP promoters recruiting these factors are associated with low protein noise in vivo [25] and why RP genes exhibit prominent transcriptional co-regulation [26],[27].

This study's more general results on complex promoter architectures primarily concern the relations between promoter structure and noise resistance. Importantly, GRF-containing architectures render promoter activity robust to influences of unspecific chromatin remodeling, independent of the compaction efficiency and speed. They maintain genes in a *poised* state for rapid (re-) activation even during prolonged absence of the main activating TF. Therefore, complex promoters can contribute much less noise to mRNA levels than simpler designs. This is particularly relevant for highly expressed and unstable proteins, where forced mRNA fluctuations dominate intrinsic noise [17] and, subsequently, total protein noise [25]. Although many GRF-containing promoters are found in highly expressed “housekeeping” genes [52], not all of these exhibit the same exceptionally low noise as RP genes in vivo [25]. This corroborates that synergistic action with ambivalent TFs such as Fhl1 is crucial.

Two important aspects warrant further investigation. First, TFs frequently interact with histones and histone (de)acetylases that co-regulate promoter activity [29],[53],[54]. Evidence from yeast indicates that dynamic recruitment of chromatin modifiers such as NuA4 can contribute to low noise [25]. Secondly, TATA boxes in promoters of many highly expressed proteins promote transcriptional re-initiation [55], but also increase intrinsic expression noise through a stable scaffold [15],[18]. Experimental data [30],[51] and our simulations demonstrate that GRF binding yields a similarly stable scaffold that leaves genes poised for transcription. Yet RP gene promoters are typically TATA-less [30], suggesting that the Rap1-Fhl-Ifh1 and similar architectures achieve high expression rates with minimal transcriptional bursts from re-initiation.

Our analysis relies on a realistic biological example and, correspondingly, some quantitative model features may be specific for the RP gene system. However, robustness analysis and model optimizations demonstrate that many stationary and qualitative dynamic features are inherent properties of the promoter structure; they are preserved within a broad range typical of physiological parameter values and TF levels. These findings may apply to structurally related promoter architectures, especially those involving certain Forkhead proteins [24],[56],[57] or ternary-complex TFs [38]. Indeed, some TCF-type promoters are characteristic of immediate-early genes in mammalian cells [38]. Performance requirements similar to RP genes hold for the synthesis of other molecular machines and for the temporal coordination of the Clb2 cluster in the yeast cell cycle [24]. These experimental observations are consistent with our proposal that the general architecture is especially suited for rapid gene (re-)activation and strong transcriptional co-regulation. We expect our study to aid in understanding complex promoter architectures not only in terms of stationary logical functions [2],[13],[14], but also regarding key qualitative aspects of gene expression dynamics.

## Materials and Methods

### Promoter Models

We modeled molecular interactions of transcription factors at the promoter using chemical reaction kinetics, which lead to ordinary differential equation (ODE) models. All deterministic simulations were performed in MATLAB 7 R14 (The MathWorks, Natick, Mass.). For stochastic simulations, we employed extended promoter models that also account for the noise in transcription factor levels, synthesis and degradation of RPmRNAs, and competitive binding of Rap1 to non-RP target genes. Stochastic simulations were performed on a PC cluster using a C-based implementation of the approximate R-leaping algorithm of Auger et al. [58]. Raw simulation results were processed and analyzed in MATLAB 7 R14 (The MathWorks, Natick, Mass.). Details regarding the chemical reaction networks, choices of kinetic constants and initial values as well as settings of the simulation algorithms are described in Protocol S1. SBML files for models 1 and 4 are provided as Protocols S2, S3, S4, S5.

### Steady-State Analysis

To assess the influence of TF levels on steady-state promoter activity, total concentrations of the TF under question were varied and the model was simulated until it reached steady state. We assigned activity levels to the resulting complexes between RP gene and TFs depending on composition (Protocol S1). We investigated the robustness of promoter activity by randomly varying the values of equilibrium constants for binding of each TF and simulating the model into steady state using measured TF concentrations [59]. For each model, 10,000 parameter sets were independently sampled from a log-uniform distribution spanning values between 10^−1^ and 10^1^ nM^−1^. Performing the same analysis for a range from 10^−2^ to 10^2^ nM^−1^ did not alter the relative sensitivity of promoter activity qualitatively (data not shown).

### Dynamic Analysis

To establish Bode-type plots for the frequency responses of the promoters, we first simulated the ODE models into steady state in the absence of the stimulant (either Ifh1 or Rap1). Subsequently, a sinusoidal input in the free stimulant concentration was applied such that the concentration oscillated between its total concentration and zero for 50 cycles at the respective frequency. For each model, 4096 points of the simulated trajectories of the relevant molecular species were used to determine their corresponding frequency, amplitude, and phase values by Fast Fourier Transformation. Despite the nonlinear nature of the models, the predominant frequency contribution to the output was always identical to the input frequency. From this data, the associated promoter activities and phase shifts between input and output were calculated.

In the stochastic simulation studies addressing RPmRNA noise for promoter designs 1 and 4, the kinetic constants of gene inactivation/reactivation were increased or decreased up to tenfold while maintaining the nominal *K_a_* value constant. Similarly, the gene inactivation rate constant was kept at its nominal value while varying the probability of an RP gene being active in the absence of TF binding between *f_a_* = 1% and *f_a_* = 99% by adjusting *K_a_* (see Protocol S1 for details). For fair comparison of mRNA noise between models (Figure 5B and 5C), we chose Ifh1 levels yielding the same mean mRNA number (45–46), corresponding to 615 (Model 1) and 1430 (Model 4) molecules of Ifh1, respectively.

### Model Validation

Details on simulation conditions, choice of experimental data, and fitting of the *IFH1* mRNA time course are described in Protocol S1.

## Supporting Information

Figure S1 Robustness of promoter activity against random perturbations in binding constants. Color-coded relative stationary promoter activity for n = 10,000 simulations of log uniformly, randomly sampled parameter combinations. (A) Model 1, as in Figure 4A, but with K_Rap1,2_ instead of K_Rap1,1_. (B) and (C) Model 2, (D) Model 3, (E) Model 4. Here, promoter activity is shown as a function of parameter K_Ifh1_. All equilibrium constants K are in units of µM^−1^.
(12.16 MB EPS)

Figure S2 Complex promoter architectures are less robust at maintaining high, but more robust in ensuring basal promoter activity. Distribution of stationary promoter activities for 10,000 vectorially-perturbed parameter sets per model. The parameters were varied according to a log uniform distribution. Distributions of those n parameter combinations out of 10,000 samples that resulted in >80% of maximal activity are shown. (A) and (B) Model 2 (n = 577), (C) and (D) Model 3 (n = 1736), (E) Model 4 (n = 2226). In (A) and (C), open symbols represent the first, filled symbols the second Rap1 binding step. In (B), open symbols represent the first, filled symbols the second Ifh1 binding step. In (D) and (E), open symbols represent Ifh1. All equilibrium constants K are in units of µM^−1^.
(0.58 MB EPS)

Figure S3 The shape of the output amplitude (promoter activity) deviates from the input shape. Phase profiles of input ((A), concentration of free Ifh1) and output ((B), relative activity of the RP promoter) for a sinusoidal input with a period of ∼17 min (f = 10^−3^ Hz). Color codes for (B): Model 1: red, Model 2: green, Model 3: blue, and Model 4: black.
(0.37 MB EPS)

Figure S4 Promoters serve as low-pass filters and differ in resistance to chromatin remodeling. Bode-type plot comparing model responses to a sinusoidal input in the concentration of free Ifh1 with (A) or without (B) gene inactivation. (A) and (B): phase shift between input and output. Color codes: Model 1 - red, Model 2 - green, Model 3 - blue, Model 4 - black. Models 3 and 4 exhibit essentially identical phase shifts for Ifh1 oscillations and hence cannot be discerned in (A). (C) and (D): normalized amplitude of promoter activity oscillations for the Rap1 containing models 1–3 in the absence (C) or presence (D) of gene silencing with free Rap1 as oscillating input. Solid vertical markers indicate the physiologically relevant frequency range.
(0.62 MB EPS)

Figure S5 Rap1-containing promoters exhibit superior input tracking in the presence of gene inactivation. (A) and (B) Promoter activity response (output) to a series of step inputs in total Ifh1 (10–100% Ifh1_T_) for the different models (Model 1 - red, Model 2 - green, Model 3 - blue, Model 4 - black) in the absence (A) and presence (B) of gene inactivation. (C) and (D) Normalized deviation between output and ideal step response shape as a function of input step height without (C) and with (D) random gene inactivation. Symbols: Model 1 - filled circles, Model 2 - open squares, Model 3 - filled triangles, Model 4 - open diamonds.
(2.76 MB EPS)

Figure S6 Rap1-containing promoters produce less noisy mRNA distributions. Stationary distributions of RP mRNA levels obtained by stochastic simulation. (A) Rap1 containing promoter (Model 1) with a mean of 46.2 mRNA molecules and CV_RPmRNA_ = 35%. (B) Simple architecture lacking Rap1 (Model 4) with a mean of 45.2 mRNA molecules and CV_RPmRNA_ = 58%. Note the markedly higher frequency of complete mRNA absence in Model 4 (B).
(0.29 MB EPS)

Figure S7 Relative changes in RP mRNA levels can depend non-monotonically on basal IFH1 expression for promoters with at least two states of nonzero transcriptional activity. Stationary RP mRNA levels before (dashed lines) and after (dash-dotted lines) stimulation of IFH1 expression (50 fold, similar to the maximum value in Figure 7A) and the corresponding fold increase (solid lines) are shown for different pre-induction values of IFH1 mRNA. All simulations were performed using the deterministic models without chromatin remodeling. (A) Model 1, (B) Model 2, (C) Model 3, and (D) Model 4.
(0.77 MB EPS)

Figure S8 Predicted TF promoter occupancies in response to stress. Comparison of simulated and measured Rap1 (upper row) and Fhl1 occupancies (lower row) under various stress conditions (experimental data from [32]). (A, D) heat shock, (B, E) osmotic shock, (C, F) rapamycin addition. Error bars indicate the standard deviation of the mean for three RP genes (RPL2B, RPL27B, and RPS11B). See Protocol S1 for details.
(0.35 MB EPS)

Protocol S1 Supporting methods
(0.88 MB DOC)

Protocol S2 SBML file for deterministic version of Model 1
(3.00 KB ZIP)

Protocol S3 SBML file for stochastic version of Model 1
(3.00 KB ZIP)

Protocol S4 SBML file for deterministic version of Model 4
(2.00 KB ZIP)

Protocol S5 SBML file for stochastic version of Model 4
(2.00 KB ZIP)
