# Supplementary material for: Precise Regulation of Gene Expression Dynamics Favors Complex Promoter Architectures
Source: PLoS Comput Biol. 2009 Jan 30;5(1):e1000279. doi: 10.1371/journal.pcbi.1000279 (PMC2615112; doi:10.1371/journal.pcbi.1000279)
Supplement: Protocol S1 — Supporting methods (0.88 MB DOC) [file pcbi.1000279.s009.doc]

# Supporting Information – Protocol S1

## Promoter Models

### Biological Justification of Wildtype Model Structure

The yeast RP gene system employs the Forkhead (FH)‑type TF Fhl1, which belongs to a family of transcriptional regulators that have an evolutionarily conserved DNA-binding motif and variable activation / repression domains [1]. Many of these TFs share the following features: (i) they can switch between a repressive and an activating form depending on interaction partners and / or protein modification state [2,3]; (ii) they affect chromatin structure by directly interacting with nucleosomes and / or by dynamically recruiting chromatin remodeling activities [1,4]; and (iii) they are direct targets of nutrient‑ and growth‑signaling pathways, or they interact with partners that are controlled by these pathways via the Forkhead-associated (FHA) domain [1,3,5]. In addition, several members of the TF class (e.g., Fkh2, Fhl1, FOXK1) require prior DNA binding by abundant general regulatory factors (GRFs) such as Rap1 or Mcm1 in yeast [3,6-8] and serum-response factor in humans [9]. They share this feature with structurally similar promoters, such as certain ternary complex factors [10,11].

High-confidence experimental datasets [12,13] show that the yeast RP gene architecture is very generic: 91 out of 137 RP genes bind Fhl1, 83 bind Ifh1, and 74 genes bind both. These data likely underestimate the true extent of binding *in vivo* [13]. Only 15 RP promoters do not bind Rap1 *in vivo*, 9 of which are predicted targets of the alternative GRFs Reb1 and Abf1 [6]. Harbison et al. [14] observed high-confidence binding (*p* < 5⋅10^-3^) of Fhl1 and of the alternative regulator Rgm1 to 106 and 3 RP genes, respectively. Hence, Rgm1 either acts indirectly or affects only a small subset of RP genes. The HMG protein Hmo1 and Fhl1 appear to bind to RP promoters cooperatively [15], but Fhl1 is stoichiometrically limiting (about 30‑fold [16]) and both single-gene deletions affect RP transcription similarly [15,17]. Additional regulators such as Sfp1 and Crf1 can contribute to RP gene regulation via Ifh1 and Fhl1 [5,18,19], but these effects are probably indirect [20] or strain-specific (Crf1). Sch9‑mediated regulation affects RP gene expression in the same qualitative fashion as Ifh1, but the mechanism of action is unknown [17,18]. However, experiments suggest that regulated binding of Ifh1 is the predominant mode of controlling promoter activity [12,13]. Therefore, a model comprising only the molecules Rap1 (representing the functionally equivalent GRFs Rap1, Abf1, and Reb1), Fhl1 (representing Fhl1 and Hmo1), and Ifh1 is expected to capture key aspects of transcriptional control of RP genes.

### Model Reactions and Kinetics

Elementary binding steps in the assembly of transcription factors at the ribosomal protein gene (RPGene) promoter were assumed to follow mass‑action laws [21,22]. Net association kinetics of two generic molecules A_i_ and B_i_ reacting according to

was hence described by

$$r_{_{i,net}}=r_{_{i,association}}-r_{i,dissociation}=k_{i}\cdot\left[A_{i}\right]\cdot\left[B_{i}\right]-k_{-i}\cdot\left[A_{i}\cdot B_{i}\right]$$

where square brackets indicate the (nuclear) concentration of the relevant species. The “⋅” in between the species names indicates formation of a protein-protein or protein‑DNA complex. From this representation, the *equilibrium binding constants* *K_i_* were directly inferred for all *i* binding steps:

$$K_{i}=\frac{k_{i}}{k_{-i}}=\frac{\left[A_{i}\cdot B_{i}\right]}{\left[A_{i}\right]\cdot\left[B_{i}\right]}\forall i$$

We assumed constant total concentrations of all species – promoter and transcription factors. The following lists of reactions provide details for all models considered, including the assignment of kinetic parameters:

#### Model 1

#### Model 2

#### Model 3

#### Model 4

#### Gene Silencing

When considering gene silencing, we added a single reaction to each promoter model. Unspecific chromatin remodeling was represented by simple first-order reactions converting an active gene (*RPGene*) into the inactive form (*RPGene_in_*) and vice versa:

### Promoter Activity and Transcriptional Efficiencies

The resulting relative transcriptional activity of the promoter was calculated as the sum of the concentrations of all promoter complexes with transcriptional activity. Fully‑assembled promoter complexes were assigned a transcriptional efficiency of *η* = 1.0 * *(*η* ∈ [0, 1]), where the definition of “fully‑assembled” was model-dependent (cf. Table S1Table S1). In this way, the maximum theoretical activity was achieved when the concentration of fully‑assembled promoters was identical to the total promoter concentration. We represented basal activity conveyed by Rap1_2_⋅RPGene complexes by choosing low values of *η*: Depending on the analysis performed, *η_basal_* was set to either 0.05 (default) or 0.2 where stated explicitly. Assignment of 5% basal activity was based on the observation that ribosomes are synthesized at about 5-10% of the normal rate in *fhl1*Δ mutants [17]. In all analyses, no qualitatively different results were obtained even if a basal relative activity of up to 20 % was assigned to these complexes unless indicated otherwise. The transcriptional efficiencies we considered for the different molecular species depending on promoter architecture are compiled in Table S1.

Table S1. Transcriptional efficiencies of different molecular complexes

| **Model Species Name** | **Transcriptional Efficiency *η*** | | | |
| --- | --- | --- | --- | --- |
|  | **Model 1** | **Model 2** | **Model 3** | **Model 4** |
| *RPGene* | 0.0 | 0.0 | 0.0 | 0.0 |
| *Rap1⋅RPGene* | 0.0 | 0.0 | 0.0 | n/a* |
| *Rap1_2_⋅RPGene* | 0.05 ‑ 0.2^#^ | 0.05 ‑ 0.2^#^ | 0.05 – 0.2^#^ | n/a |
| *Fhl1⋅Rap1_2_⋅RPGene* | 0.0 | n/a | n/a | n/a |
| *Ifh1⋅Fhl1⋅Rap1_2_⋅RPGene* | 1.0 | n/a | n/a | n/a |
| *Ifh1⋅Rap1_2_⋅RPGene* | n/a | 0.0 | 1.0 | n/a |
| *Ifh1_2_⋅Rap1_2_⋅RPGene* | n/a | 1.0 | n/a | n/a |
| *Ifh1 RPGene* | n/a | n/a | n/a | 1.0 |

* n/a: not applicable due to model structure.

^#^: depending on setting for model analysis

### Total Concentrations

All concentrations were calculated assuming an exclusively nuclear localization of all molecules and a nuclear volume of about 2 fL (for a nuclear diameter of about 1.5 µm [23]) for 70 fL total cell volume of a haploid cell [24]. Protein abundances were taken from [16]: 9206 molecules Rap1 (lumped species Rap1 and Abf1), 639 molecules Fhl1, and 1431 molecules Ifh1. The concentration of ribosomal protein genes was calculated by relating their total number per haploid cell (138, see [17]) to the nuclear volume.

### Kinetic Parameters

Kinetic parameters of the different association and dissociation reactions were either taken from *in vitro* measurements or inferred based on plausibility constraints and experimental data. This included, in particular, the requirement that the RP gene promoter should exhibit near maximum stationary activity (chosen cutoff ≥ 80%) for TF concentrations measured under optimal growth conditions because promoters of highly transcribed genes tend to be active at relatively higher induction levels [25,26]. This criterion was used to scan the parameter space of equilibrium binding constants *K_i_* for each binding step in every model in the range of $\lg K_{i}=n$ with $n\in\left\{-1,0,1\right\}$*.* In this way, we generated all feasible combinations of equilibrium constant values and determined the associated stationary promoter activity through simulation. Allowing *n* to assume values of -2 or 2 did not result in significant qualitative changes concerning both, the absolute values of promoter activity reached at low and high TF concentrations and the shape of the dose‑response curves, respectively. Hence, the search was restricted to the smaller parameter region. Among the parameter sets fulfilling the cutoff criterion, we selected the one providing the highest activity (Model 4) or a representative set within 1% of the highest relative promoter activity attained by the best combination for each model (models 1-3). In the latter case, we selected a parameter set with different binding affinities for the two Rap1 binding steps in agreement with measured data [27] and chose sets with identical values for *K_Rap1,1_* and *K_Rap1,2_* in models 1-3 to allow for fair model comparison.

Given the measured half lives of Rap1-DNA complexes, knowledge of the equilibrium constants is sufficient to calculate the corresponding kinetic association constants *k_1_* and *k_2_* in models 1-3 (see Table S2.). Since occupation of RP gene promoters by both Rap1 and Fhl1 is essentially unresponsive to changes in nutrient status [12,17,28], we assumed the same half life of the Fhl1⋅Rap1_2_⋅RPGene complex as measured for the Rap1_2_⋅RPGene complex (6 min) [27]. By contrast, we chose a threefold lower half life (2 min) for the Ifh1⋅Fhl1⋅Rap1_2_⋅RPGene complex to account for the rapid loss of Ifh1 from of promoters [13]. With these data and the previously selected equilibrium constants, all remaining kinetic constants in Model 1 could be computed. The kinetic constants in models 2-4 were assigned the same values as the corresponding binding steps in Model 1. For Model 2, both Ifh1 binding steps were assumed to possess the same binding kinetics. The results of the robustness analysis (cf. Figure 3, Figure S1, and Figure S2) essentially confirmed the sensibility of the chosen equilibrium binding constants.

We described the impact of unspecific chromatin modifying activities on promoter behavior by a reversible and constitutive process that maintains a compact chromatin state (assembled nucleosomes) in the absence of TF binding. In the models, TFs can bind only to genes in the open conformation, thereby removing them from the pool of genes available for compaction. Importantly, we do not assume recruitment of chromatin modifiers through bound TFs; such chromatin modification is considered as integral to promoter activation and, hence, implicitly represented by the corresponding time constants. For the dynamics of gene inactivation / reactivation due to gene silencing, we estimated the inactivation velocity *k_-5_* using H4 acetylation patterns of *RPL9A* during rapamycin-mediated inhibition of Esa1 [29]. Data for Esa1 action at yeast *SUC2* [30] imply that histone modifiers can significantly speed up chromatin remodeling ‑ about fivefold in this particular case ‑ by stimulating efficient binding of remodeling complexes. Therefore, we assumed a fivefold reduced velocity of the (de)acetylation processes in the absence of TFs compared to the situation with efficiently recruited modifiers. The rate constant *k_5_* of reactivation (indices correspond to Model 1) was then calculated from

$$k_{5}=K_{a,5}\cdot k_{-5}$$

where the equilibrium binding constant *K_a,5_* is determined by the assumed fraction of active genes *f_a_* (in %) in the absence of any transcription factor binding

$$K_{a,5}=\frac{f_{a}}{100\%-f_{a}}$$

We used a nominal value of *f_a_* = 10% (*K_a,5_* = 0.11) for our simulations unless noted otherwise. This value likely is a conservative underestimate of the repression efficiency, thereby providing a lower bound for the expected effect of gene silencing.

Table S2. Kinetic parameters for deterministic simulations of alternative promoters

| **Model 1** | **Model 2** | **Model 3** | **Model 4** | **Value** | **Unit** | **Reference** |
| --- | --- | --- | --- | --- | --- | --- |
| *k_1_* | *k_1_* | *k_1_* |  | 1.44⋅10^-3^ | µM^-1^⋅s^-1^ | inferred |
| *k-_1_* | *k-_1_* | *k-_1_* |  | 1.44⋅10^-4^ | s^-1^ | [27] |
| *k_2_* | *k_2_* | *k_2_* |  | 1.93⋅10^-3^ | µM^-1^⋅s^-1^ | inferred |
| *k-_2_* | *k-_2_* | *k-_2_* |  | 1.93⋅10^-3^ | s^-1^ | [27] |
| *k_3_* |  |  |  | 1.93⋅10^-2^ | µM^-1^⋅s^-1^ | inferred |
| *k-_3_* |  |  |  | 1.93⋅10^-3^ | s^-1^ | inferred |
| *k_4_* | *k_3_,k_4_* | *k_3_* | *k_1_* | 5.78⋅10^-2^ | µM^-1^⋅s^-1^ | inferred |
| *k-_4_* | *k-_3_, k-_4_* | *k-_3_* | *k-_1_* | 5.78⋅10^-3^ | s^-1^ | inferred |
| *k_5_* | *k_5_* | *k_4_* | *k_2_* | 2.98⋅10^-4^ | s^-1^ | inferred from  Rohde & Cardenas [29] |
| *k-_5_* | *k-_5_* | *k-_4_* | *k-_2_* | 3.31⋅10^-5^ | s^-1^ | inferred |

### Optimization of Individual Models

Kinetic parameters of the individual models were optimized with respect to a specific objective function (cf. below) using a custom-coded Evolutionary Strategy as described previously [31]. Kinetic parameters for association and dissociation rates were allowed to vary over a wide range (10^-4^ ‑ 1 µM^-1^s^-1^ for *k_on_*,  10^-5^ – 0.1 s^-1^ for *k_off_*) based on values reported in the literature [27,32] and including TF affinities in the typical nanomolar to micromolar range [33]. For optimizations in the presence of gene inacitvation, kinetic constants for the gene inactivation were left constant at their nominal values (cf. Table S2) or set to zero otherwise. Three different objective functions were investigated:

a) simultaneous maximization of stationary promoter activity in the presence and
minimization in the absence of Ifh1

b) as in a), but with the additional requirement that promoter shut-off in the absence of Ifh1
should not only be efficient, but also rapid

c) minimization of the summed activity output deviation from an ideal step response
(instantaneous step output) for a series of step changes in total Ifh1 concentration

For variant c), the individual step responses were normalized to the respective final steady state activity value to permit fair comparison of different step heights. Also, a combination of variant c) and maximization of stationary activity for the largest input step was assayed. All models were optimized in both the presence and absence of gene inactivation, respectively, for each objective function variant. At least three independent runs of at least 600 generations with a (*µ* +* λ*) strategy (*µ* = 2 parents, *λ* = 8 children) were performed for each combination of model/objective function, using different random starting points in parameter space for each run.

In all cases investigated, the model without Rap1 (Model 4) provided inferior performance compared to architectures containing Rap1 (esp. Model 1 and Model 3) if gene silencing was present (Figure S5 and data not shown). Convergence to the same objective function value (within seven significant digits) was attained reproducibly in all runs of an optimization set. Throughout, certain optimal parameter values (esp. for Rap1 binding) would vary between runs with no significant impact on objective function value whereas essentially unique values resulted for the last Ifh1 binding step, thus further supporting the results of the robustness analysis (see Tables S3 - S5 for details).

**Table S3.** Optimal parameters for maximum stationary promoter activity without (top) and with (bottom) gene inactivation.

| **Parameter** | **Unit** | **Model 1** | | **Model 2** | | **Model 3** | | **Model 4** | |
| --- | --- | --- | --- | --- | --- | --- | --- | --- | --- |
|  |  | **Mean** | **CV (%)** | **Mean** | **CV (%)** | **Mean** | **CV (%)** | **Mean** | **CV (%)** |
| **kRa1_on** | µM^-1^⋅s^-1^ | 3.91E-01 | 95 | 1.20E-01 | 168 | 3.88E-01 | 135 | - | - |
| **kRa1_off** | s^-1^ | 3.49E-02 | 160 | 9.99E-02 | 0.03 | 2.34E-02 | 172 | - | - |
| **kRa2_on** | µM^-1^⋅s^-1^ | 6.16E-01 | 83 | 9.98E-04 | 69 | 5.12E-03 | 122 | - | - |
| **kRa2_off** | s^-1^ | 1.82E-02 | 90 | 1.00E-01 | 0.00 | 9.59E-02 | 6.0 | - | - |
| **kFhl1_on** | µM^-1^⋅s^-1^ | 1.00E+00 | 0.01 | - | - | - | - | - | - |
| **kFhl1_off** | s^-1^ | 1.00E-04 | 0.00 | - |  | - | - | - | - |
| **kIfh1_1_on** | µM^-1^⋅s^-1^ | 1.00E+00 | 0.00 | 1.00E+00 | 0.00 | 1.00E+00 | 0.00 | 1.00E+00 | 0.00 |
| **kIfh1_1_off** | s^-1^ | 8.08E-04 | 0.02 | 1.00E-04 | 0.00 | 6.06E-04 | 0.07 | 8.20E-04 | 0.08 |
| **kIfh1_2_on** | µM^-1^⋅s^-1^ | - | - | 1.00E+00 | 0.00 | - | - | - | - |
| **kIfh1_2_off** | s^-1^ | - | - | 8.93E-04 | 0.17 | - | - | - | - |
| **Final objective function value** | - | 7.68E-04 | 0.01 | 1.76E-03 | 1.6 | 1.09E-02 | 0.87 | 7.67E-04 | 0.00 |
| **max activity** | % | 99.93 | 0.00 | 99.92 | 0.00 | 99.49 | 0.00 | 99.93 | 0.00 |
| **min activity** | % | 0.002 | 0.00 | 0.08 | 3.45 | 0.52 | 1.27 | 0.001 | 1.3 |
| **Ka_Ra1** | µM^-1^ | 2304 | 172 | 1.21 | 168 | 3358 | 134 | - | 0.00 |
| **Ka_Ra2** | µM^-1^ | 70 | 86 | 0.010 | 69 | 0.052 | 120 | - | 0.00 |
| **Ka_Fh** | µM^-1^ | 9999 | 0.01 | - | - | - | - | - | - |
| **Ka_If1** | µM^-1^ | 1237 | 0.02 | 10000 | 0.00 | 1649 | 0.07 | 1219 | 0.08 |
| **Ka_If2** | µM^-1^ | - | - | 1120 | 0.17 | - | - | - | - |

| **Parameter** | **Unit** | **Model 1** | | **Model 2** | | **Model 3** | | **Model 4** | |
| --- | --- | --- | --- | --- | --- | --- | --- | --- | --- |
|  |  | **Mean** | **CV (%)** | **Mean** | **CV (%)** | **Mean** | **CV (%)** | **Mean** | **CV (%)** |
| **kRa1_on** | µM^-1^⋅s^-1^ | 9.99E-01 | 0.08 | 1.00E+00 | 0.01 | 1.00E+00 | 0.00 | - | - |
| **kRa1_off** | s^-1^ | 1.72E-04 | 51 | 1.00E-04 | 0.04 | 1.00E-04 | 0.13 | - | - |
| **kRa2_on** | µM^-1^⋅s^-1^ | 5.21E-01 | 83 | 2.28E-04 | 2.6 | 1.44E-03 | 19 | - | - |
| **kRa2_off** | s^-1^ | 7.90E-02 | 45 | 1.00E-01 | 0.00 | 8.99E-02 | 19 | - | - |
| **kFhl1_on** | µM^-1^⋅s^-1^ | 1.00E+00 | 0.00 | - | - | - | - | - | - |
| **kFhl1_off** | s^-1^ | 1.00E-04 | 0.01 | - | - | - | - | - | - |
| **kIfh1_1_on** | µM^-1^⋅s^-1^ | 1.00E+00 | 0.00 | 1.00E+00 | 0.00 | 1.00E+00 | 0.00 | 1.00E+00 | 0.00 |
| **kIfh1_1_off** | s^-1^ | 8.09E-04 | 0.06 | 4.40E-04 | 92 | 6.07E-04 | 0.03 | 7.15E-04 | 0.02 |
| **kIfh1_2_on** | µM^-1^⋅s^-1^ | - | - | 1.00E+00 | 0.00 | - | - | - | - |
| **kIfh1_2_off** | s^-1^ | - | - | 8.16E-04 | 5.1 | - | - | - | - |
| **Final objective function value** | - | 8.01E-04 | 0.01 | 1.70E-03 | 2.2 | 1.09E-02 | 0.00 | 2.24E-03 | 0.00 |
| **max activity** | % | 99.93 | 0.00 | 99.93 | 0.00 | 99.49 | 0.00 | 99.77 | 0.00 |
| **min activity** | % | 0.002 | 0.21 | 0.08 | 1.1 | 0.52 | 0.05 | 0.002 | 0.21 |
| **Ka_Ra1** | µM^-1^ | 6755 | 43 | 10000 | 0.04 | 9989 | 0.13 | - | - |
| **Ka_Ra2** | µM^-1^ | 5.8 | 61 | 0.0023 | 2.6 | 0.016 | 0.07 | - | - |
| **Ka_Fh** | µM^-1^ | 9998 | 0.004 | - | - | - | - | - | - |
| **Ka_If1** | µM^-1^ | 1236 | 0.06 | 2275 | 60 | 1648 | 0.03 | 1398 | 0.02 |
| **Ka_If2** | µM^-1^ | - | - | 1226 | 5.3 | - | - | - | - |

**Table S4.** Optimal parameters for maximum stationary promoter activity and fast shut-off and minimum activity in the absence of Ifh1 (top: without gene inactivation; bottom: with gene inactivation).

| **Parameter** | **Unit** | **Model 1** | | **Model 2** | | **Model 3** | | **Model 4** | |
| --- | --- | --- | --- | --- | --- | --- | --- | --- | --- |
|  |  | **Mean** | **CV (%)** | **Mean** | **CV (%)** | **Mean** | **CV (%)** | **Mean** | **CV (%)** |
| **kRa1_on** | µM^-1^⋅s^-1^ | 5.74E-01 | 83 | 3.12E-01 | 171 | 6.82E-01 | 57 | - | - |
| **kRa1_off** | s^-1^ | 3.71E-02 | 56 | 6.39E-02 | 69.65 | 3.39E-02 | 167 | - | - |
| **kRa2_on** | µM^-1^⋅s^-1^ | 4.88E-01 | 71 | 8.30E-04 | 65 | 6.82E-03 | 1 | - | - |
| **kRa2_off** | s^-1^ | 1.30E-02 | 104 | 1.00E-01 | 0.01 | 1.00E-01 | 0.0 | - | - |
| **kFhl1_on** | µM^-1^⋅s^-1^ | 9.98E-01 | 0.22 | - | - | - | - | - | - |
| **kFhl1_off** | s^-1^ | 1.00E-04 | 0.28 | - | - | - | - | - | - |
| **kIfh1_1_on** | µM^-1^⋅s^-1^ | 1.00E+00 | 0.00 | 1.00E+00 | 0.02 | 1.00E+00 | 0.00 | 1.00E+00 | 0.00 |
| **kIfh1_1_off** | s^-1^ | 1.10E-02 | 0.09 | 1.00E-04 | 0.01 | 6.27E-03 | 0.07 | 1.11E-02 | 0.04 |
| **kIfh1_2_on** | µM^-1^⋅s^-1^ | - | - | 1.00E+00 | 0.00 | - | - | - | - |
| **kIfh1_2_off** | s^-1^ | - | - | 1.11E-02 | 0.05 | - | - | - | - |
| **Final objective function value** | - | 1.83E-02 | 0.00 | 1.88E-02 | 0.1 | 4.77E-02 | 0.00 | 1.83E-02 | 0.00 |
| **max activity** | % | 99.08 | 0.00 | 99.07 | 0.00 | 98.40 | 0.00 | 99.08 | 0.00 |
| **min activity** | % | 0.001 | 0.39 | 0.06 | 3.07 | 1.61 | 0.14 | 0.000 | 40.1 |
| **Ka_Ra1** | µM^-1^ | 13 | 68 | 24.76 | 173 | 503 | 152 | - | - |
| **Ka_Ra2** | µM^-1^ | 425 | 159 | 0.008 | 65 | 0.068 | 1 | - | - |
| **Ka_Fh** | µM^-1^ | 9953 | 0.39 | - | - | - | - | - | - |
| **Ka_If1** | µM^-1^ | 91 | 0.09 | 9997 | 0.03 | 159 | 0.07 | 90 | 0.04 |
| **Ka_If2** | µM^-1^ | - | - | 90 | 0.05 | - | - | - | - |

| **Parameter** | **Unit** | **Model 1** | | **Model 2** | | **Model 3** | | **Model 4** | |
| --- | --- | --- | --- | --- | --- | --- | --- | --- | --- |
|  |  | **Mean** | **CV (%)** | **Mean** | **CV (%)** | **Mean** | **CV (%)** | **Mean** | **CV (%)** |
| **kRa1_on** | µM^-1^⋅s^-1^ | 1.00E+00 | 0.02 | 9.99E-01 | 0.05 | 9.99E-01 | 0.05 | - | - |
| **kRa1_off** | s^-1^ | 2.71E-03 | 85 | 1.00E-04 | 0.33 | 1.04E-04 | 2.84 | - | - |
| **kRa2_on** | µM^-1^⋅s^-1^ | 3.65E-01 | 130 | 2.41E-04 | 0.3 | 4.78E-03 | 71.43 | - | - |
| **kRa2_off** | s^-1^ | 8.75E-03 | 102 | 1.00E-01 | 0.01 | 7.07E-02 | 71.56 | - | - |
| **kFhl1_on** | µM^-1^⋅s^-1^ | 9.96E-01 | 0.61 | - | - | - | - | - | - |
| **kFhl1_off** | s^-1^ | 1.00E-04 | 0.03 | - | - | - | - | - | - |
| **kIfh1_1_on** | µM^-1^⋅s^-1^ | 1.00E+00 | 0.00 | 1.00E+00 | 0.03 | 1.00E+00 | 0.00 | 1.00E+00 | 0.00 |
| **kIfh1_1_off** | s^-1^ | 1.10E-02 | 0.10 | 1.00E-04 | 0 | 6.27E-03 | 0.23 | 5.93E-03 | 0.04 |
| **kIfh1_2_on** | µM^-1^⋅s^-1^ | - | - | 1.00E+00 | 0.00 | - | - | - | - |
| **kIfh1_2_off** | s^-1^ | - | - | 1.11E-02 | 0.2 | - | - | - | - |
| **Final objective function value** | - | 1.83E-02 | 0.00 | 1.89E-02 | 0.0 | 4.78E-02 | 0.22 | 3.42E-02 | 0.00 |
| **max activity** | % | 99.08 | 0.00 | 99.07 | 0.00 | 98.40 | 0.00 | 97.76 | 0.00 |
| **min activity** | % | 0.001 | 0.63 | 0.06 | 0.2 | 1.61 | 0.39 | 0.000 | 36.69 |
| **Ka_Ra1** | µM^-1^ | 953 | 121 | 9946 | 0.38 | 9649 | 2.94 | - | - |
| **Ka_Ra2** | µM^-1^ | 33.3 | 39 | 0.0024 | 0.3 | 0.068 | 0.57 | - | - |
| **Ka_Fh** | µM^-1^ | 9950 | 0.633 | - | - | - | - | - | - |
| **Ka_If1** | µM^-1^ | 91 | 0.10 | 9997 | 0 | 159 | 0.23 | 169 | 0.04 |
| **Ka_If2** | µM^-1^ | - | - | 90 | 0.2 | - | - | - | - |

**Table S5.** Optimal parameters for minimum deviation from ideal step response shape (step function) in the presence of gene inactivation (top: optimized for ideal shape; bottom: optimized for ideal shape and maximum stationary activity at the maximum Ifh1 concentration).

| **Parameter** | **Unit** | **Model 1** | | **Model 2** | | **Model 3** | | **Model 4** | |
| --- | --- | --- | --- | --- | --- | --- | --- | --- | --- |
|  |  | **Mean** | **CV (%)** | **Mean** | **CV (%)** | **Mean** | **CV (%)** | **Mean** | **CV (%)** |
| **kRa1_on** | µM^-1^⋅s^-1^ | 9.93E-01 | 0.49 | 9.92E-01 | 1.21 | 9.97E-01 | 0.22 | - | - |
| **kRa1_off** | s^-1^ | 1.18E-02 | 90 | 2.32E-04 | 42 | 3.63E-03 | 46 | - | - |
| **kRa2_on** | µM^-1^⋅s^-1^ | 5.53E-01 | 71 | 5.45E-01 | 49 | 8.43E-01 | 5.05 | - | - |
| **kRa2_off** | s^-1^ | 1.97E-02 | 104 | 1.03E-04 | 2.02 | 2.67E-04 | 42 | - | - |
| **kFhl1_on** | µM^-1^⋅s^-1^ | 9.54E-01 | 5.05 | - | - | - | - | - | - |
| **kFhl1_off** | s^-1^ | 1.07E-04 | 8.09 | - | - | - | - | - | - |
| **kIfh1_1_on** | µM^-1^⋅s^-1^ | 1.00E+00 | 0.00 | 1.00E+00 | 0.00 | 1.00E+00 | 0.00 | 8.41E-03 | 0.03 |
| **kIfh1_1_off** | s^-1^ | 1.00E-01 | 0.00 | 1.00E-01 | 0.00 | 1.00E-01 | 0.00 | 1.00E-01 | 0.00 |
| **kIfh1_2_on** | µM^-1^⋅s^-1^ | - | - | 1.02E-05 | 2.96 | - | - | - | - |
| **kIfh1_2_off** | s^-1^ | - | - | 9.95E-02 | 0.90 | - | - | - | - |
| **Final objective function value** | - | 1.80E+00 | 0.01 | 1.09E+00 | 0.05 | 1.11E+00 | 0.01 | 8.32E+01 | 0.00 |
| **Ka_Ra1** | µM^-1^ | 531 | 153 | 5022 | 53 | 340 | 63 | - | - |
| **Ka_Ra2** | µM^-1^ | 38 | 94 | 5333 | 51 | 3568 | 40 | - | - |
| **Ka_Fh** | µM^-1^ | 8955 | 4.81 | - | - | - | - | - | - |
| **Ka_If1** | µM^-1^ | 10 | 0.00 | 10 | 0.00 | 10 | 0.00 | 0.084 | 0.03 |
| **Ka_If2** | µM^-1^ | - | - | 0.0001 | 3.89 | - | - | - | - |

| **Parameter** | **Unit** | **Model 1** | | **Model 2** | | **Model 3** | | **Model 4** | |
| --- | --- | --- | --- | --- | --- | --- | --- | --- | --- |
|  |  | **Mean** | **CV (%)** | **Mean** | **CV (%)** | **Mean** | **CV (%)** | **Mean** | **CV (%)** |
| **kRa1_on** | µM^-1^⋅s^-1^ | 8.91E-01 | 12 | 1.00E+00 | 0.00 | 1.00E+00 | 0.00 | - | - |
| **kRa1_off** | s^-1^ | 1.96E-02 | 87 | 1.66E-04 | 68 | 1.01E-04 | 0.65 | - | - |
| **kRa2_on** | µM^-1^⋅s^-1^ | 8.10E-01 | 20 | 9.42E-01 | 5.78 | 6.96E-01 | 55 | - | - |
| **kRa2_off** | s^-1^ | 6.77E-02 | 56 | 2.85E-02 | 124.39 | 1.60E-02 | 77 | - | - |
| **kFhl1_on** | µM^-1^⋅s^-1^ | 9.04E-01 | 14 | - | - | - | - | - | - |
| **kFhl1_off** | s^-1^ | 1.05E-03 | 55 | - | - | - | - | - | - |
| **kIfh1_1_on** | µM^-1^⋅s^-1^ | 1.00E+00 | 0.00 | 1.00E+00 | 0.00 | 1.00E+00 | 0.00 | 1.00E+00 | 0.00 |
| **kIfh1_1_off** | s^-1^ | 1.00E-04 | 0.02 | 1.08E-04 | 5.73 | 1.00E-04 | 0.00 | 1.00E-04 | 0.00 |
| **kIfh1_2_on** | µM^-1^⋅s^-1^ | - | - | 1.00E+00 | 0.00 | - | - | - | - |
| **kIfh1_2_off** | s^-1^ | - | - | 1.00E-04 | 0.01 | - | - | - | - |
| **Final objective function value** | - | 1.09E+03 | 0.14 | 1.06E+03 | 0.51 | 1.03E+03 | 0.02 | 8.27E+05 | 0.00 |
| **Ka_Ra1** | µM^-1^ | 66 | 55 | 7741 | 49 | 9951 | 0.65 | - | - |
| **Ka_Ra2** | µM^-1^ | 10 | 12 | 1046 | 168 | 55 | 43 | - | - |
| **Ka_Fh** | µM^-1^ | 849 | 28 | - | - | - | - | - | - |
| **Ka_If1** | µM^-1^ | 9999 | 0.01 | 9293 | 5.60 | 10000 | 0.00 | 10000 | 0.00 |
| **Ka_If2** | µM^-1^ | - | - | 9999 | 0.01 | - | - | - | - |

Stochastic Simulations

### Stochastic Models and Extensions

# Simulating the above promoter models in a stochastic scenario requires conversion of the corresponding kinetic constants from the deterministic representation to a stochastic one. In the absence of reactions involving the multimerization of identical reactands – which applies to all models considered here ‑ this conversion can be computed according to

$$k_{j}=\frac{\tilde{k}_{j}}{\left(N_{A}\cdot V\right)^{-1-\sum_{i} \nu_{ij}}}$$

Here, $k_{j}$ stands for the stochastic reaction constant referring to absolute molecule numbers and $\tilde{k}_{j}$ is the equivalent deterministic constant in terms of a molar representation. *N_A_* denotes *Avogadro’s number* (6.022∙10^23^ molecules/mol), *ν_ij_* is the *stoichiometric coefficient* of the molecular species *i* in reaction channel *j*, and $-\sum_{i} \nu_{ij}$ represents the *molecularity* of reaction *j*. The molecularity is given by the negative sum of reactand reaction orders considering negative stoichiometric coefficients for educts. For example, for an irreversible reaction

with $r_{j}=k_{j}\cdot n_{A}\cdot n_{B}^{2}$ we obtain $-\sum_{i} \nu_{ij}=-\text{(}-1-2\text{)}=3$. For efficient conversion of kinetic parameters from the deterministic to the stochastic representation we adapted published MATLAB scripts [34].

In addition, we accounted for the noise in transcription factor levels (*n_TFi_*) by incorporating stochastic descriptions for production (index *TL*) and decay (index *D*) of transcription factors and their associated mRNAs (indices *TC* and *D*), respectively.

$$\frac{dn_{TF_{i}mRNA}}{dt}=a_{TC,TF_{i}mRNA,syn}-a_{DEG,TF_{i}mRNA}$$

$$\frac{dn_{TF_{i}}}{dt}=a_{TL,TF_{i}}-a_{D,TF_{i}}$$

with *TF_i_* = *Rap1*, *Fhl1*, and *Ifh1*, respectively. The corresponding *propensities a* for the degradation reactions are given by:

$$a_{DEG,TF_{i}mRNA}=k_{DEG,TF_{i}mRNA}\cdot n_{TF_{i}mRNA}$$

$$a_{D,TF_{i}}=k_{D,TF_{i}}\cdot n_{TF_{i}}$$

where the first‑order degradation constants were calculated from the large‑scale datasets for apparent half lives of yeast mRNA [35] and proteins [36]. Since under stationary conditions (index *0*) the propensities of synthesis must equal those of degradation, we used the stationary mRNA numbers for the TFs measured by Holstege et al. [35] and the protein numbers reported by Ghaemmaghami et al. [16] to determine the synthesis propensities from

$$a_{TC,TF_{i}mRNA}=k_{TC,TF_{i}mRNA}=k_{DEG,TF_{i}mRNA}\cdot n_{TF_{i}mRNA}^{0}=const.$$

and

$$a_{TL,TF_{i}}=k_{TL,TF_{i}}\cdot n_{TF_{i}mRNA}^{0}\cdot n_{Ribosomes}^{0}=const.$$

with

$$k_{TL,TF_{i}}=\frac{k_{D,TF_{i}}\cdot n_{TF_{i}}^{0}}{n_{TF_{i}mRNA}^{0}\cdot n_{Ribosomes}^{0}}$$

In addition, we considered 226 non-RP genes with assumed dimeric binding sites to account for competitive binding of Rap1 at other target genes in Model 1. Synthesis and degradation of RPmRNAs were modeled similar to that of TFmRNAs:

$$\frac{dn_{RP_{i}mRNA}}{dt}=a_{TC,RP_{i}mRNA}-a_{DEG,RP_{i}mRNA}$$

with

$$a_{DEG,RP_{i}mRNA}=k_{DEG,RPmRNA}\cdot n_{RP_{i}mRNA}$$

and

$$a_{TC,RP_{i}mRNA}=k_{TC}\cdot\eta_{act.RP_{i}Gene}\cdot n_{act.RP_{i}Gene}\cdot n_{RNAPolII}^{0}$$

where $n_{act.RP_{i}Gene}$ and $n_{act.RP_{i}Gene}$ denote the amount of a specific active TF‑RPGene promoter complex and of RNA polymerase II, respectively. The transcriptional efficiency of the corresponding promoter complex is represented by $\eta_{act.RP_{i}Gene}$ (cf. Table S1). In the simulations, we treated three representative RP genes as individual species. The remaining 135 RP genes were considered as lumped species that compete with the individual RP genes for TFs, but for which synthesis and degradation of RPmRNAs was not modeled. An overview of the stochastic kinetic constants is provided i Table S6. Table S6; initial values for newly introduced molecular species are given in Table S7.

Total TF levels cannot be set exactly by adjusting the synthesis and degradation rates of the free TF pools because a significant fraction of TF can be bound to promoters (see below). To more closely reproduce the total TF numbers from experimental data [16], we not only considered degradation of free TF pools, but also degradation of selected TF‑RPGene complexes. Reasoning that access of degrading activities to TFs would be limited in complexes, we modeled TF degradation only for those molecular species, where the corresponding TF represented the last addition to a TF-RPGene complex. For example, *Rap1* can be degraded in $Rap1\cdot RPGene$ and $Rap1_{2}\cdot RPGene$, but not in $Fhl1\cdot Rap1_{2}\cdot RPGene$ or $Ifh1\cdot Fhl1\cdot Rap1_{2}\cdot RPGene$. This way, stationary total TF levels differed by at most 3 % and usually less from measured data. The good agreement occurs because the majority of Rap1 and Fhl1 remain free in all scenarios and because the model describes total Ifh1 degradation exactly.

Table S6. Kinetic parameters used for stochastic simulations (nominal setting)

| **Model 1** | **Model 4** | **Value** | **Unit** | **Reference** |
| --- | --- | --- | --- | --- |
| *Core Promoter Models* | | | | |
| *k_a,Rap1nuc1_* (*k_1_*) |  | 1.20⋅10^-6^ | molecules^-1^⋅s^-1^ | inferred |
| *k_d,Rap1nuc1_* (*k_-1_*) |  | 1.44⋅10^-4^ | s^-1^ | [27] |
| *k_a,Rap1nuc2_* (*k_2_*) |  | 1.61⋅10^-6^ | molecules^-1^⋅s^-1^ | inferred |
| *k_d,Rap1nuc2_* (*k_-2_*) |  | 1.93⋅10^-3^ | s^-1^ | [27] |
| *k_a,Fhl1nuc_* (*k_3_*) |  | 1.61⋅10^-5^ | molecules^-1^⋅s^-1^ | inferred |
| *k_d,Fhl1nuc_* (*k_-3_*) |  | 1.93⋅10^-3^ | s^-1^ | inferred |
| *k_a,Ifh1nuc_* (*k_4_*) | *k_a,Ifh1nuc_* (*k_1_*) | 4.83⋅10^-5^ | molecules^-1^⋅s^-1^ | inferred |
| *k_d,Ifh1nuc_* (*k_-4_*) | *k_d,Ifh1nuc_* (*k_-1_*) | 5.78⋅10^-3^ | s^-1^ | inferred |
| *k_in,G_* (*k_5_*) | *k_in,G_* (*k_2_*) | 2.98⋅10^-4^ | s^-1^ | inferred from [29] |
| *k_act,Gin_* (*k_-5_*) | *k_act,Gin_* (*k_-2_*) | 3.31⋅10^-5^ | s^-1^ | inferred |
| *RPmRNA Synthesis and Degradation* | | | | |
| *k_TC_* | *k_TC_* | 1.36⋅10^-5^ | molecules ^-1^⋅s^-1^ | effective value computed from experimental data* according to $k_{TC}=K_{ass}\cdot k_{TC,elong}\cdot l_{RPmRNA}^{-1}$ |
| *k_DEG,RPmRNA_* | *k_DEG,RPmRNA_* | 1.16⋅10^-3^ | s^-1^ | inferred from [35] |
| *TF Synthesis and Degradation* | | | | |
| *k_TC,Rap1mRNA_* | *k_TC,Rap1mRNA_* | 8.67⋅10^-4^ | molecules^-1^⋅s^-1^ | inferred from [35] |
| *k_TC,Fhl1mRNA_* | *k_TC,Fhl1mRNA_* | 4.62⋅10^-4^ | molecules^-1^⋅s^-1^ | inferred from [35] |
| *k_TC,Ifh1mRNA_* | *k_TC,Ifh1mRNA_* | 4.81⋅10^-4^ | molecules^-1^⋅s^-1^ | inferred from [35] |
| *k_TL,Rap1mRNA_* | *k_TL,Rap1mRNA_* | 1.23⋅10^-5^ | molecules^-1^⋅s^-1^ | inferred |
| *k_TL,Fhl1mRNA_* | *k_TL,Fhl1mRNA_* | 1.36⋅10^-6^ | molecules^-1^⋅s^-1^ | inferred |
| *k_TL,Ifh1mRNA_* | *k_TL,Ifh1mRNA_* | 1.18⋅10^-6^ | molecules^-1^⋅s^-1^ | inferred |
| *k_DEG,Rap1mRNA_* | *k_DEG,Rap1mRNA_* | 9.63⋅10^-4^ | s^-1^ | [35] |
| *k_DEG,Fhl1mRNA_* | *k_DEG,Fhl1mRNA_* | 7.70⋅10^-4^ | s^-1^ | [35] |
| *k_DEG,Ifh1mRNA_* | *k_DEG,Ifh1mRNA_* | 4.81⋅10^-4^ | s^-1^ | [35] |
| *k_D,Rap1_* | *k_D,Rap1_* | 2.75⋅10^-4^ | s^-1^ | [36] |
| *k_D,Fhl1_* | *k_D,Fhl1_* | 2.89⋅10^-4^ | s^-1^ | [36] |
| *k_D,IFH1_* | *k_D,IFH1_* | 7.45⋅10^-4^ | s^-1^ | [36] |

*: *k_TC,elong_*: transcriptional elongation rate of 33 nucleotides per second [37], *K_ass_* : assumed association constant of *K_a_* = 0.5 µM_nuc_ for the promoter‑*RNAPolII* complex, and *l_RPmRNA_* : length of an average ribosomal protein mRNA (1015 nucleotides as inferred from the data of Hurowitz and Brown [38]).

Table S7. Initial values of additional molecular species in the stochastic model

| **Molecular Species** | **Amount** | **Unit** | **Reference** |
| --- | --- | --- | --- |
| *RAP1 Gene* | 1 | molecules / cell | SGD Database [39] |
| *FHL1 Gene* | 1 | molecules / cell | SGD Database |
| *IFH1 Gene* | 1 | molecules / cell | SGD Database |
| *RAP1* mRNA | 0.9* | molecules / cell | [35] |
| *FHL1* mRNA | 0.6* | molecules / cell | [35] |
| *IFH1* mRNA | 1.0* | molecules / cell | [35] |
| *RPmRNA* | 60 | molecules / cell | using Rpl1B as example [35] |
| *Constant Species* | | | |
| *Ribosomes* | 2.26⋅10^5^ | molecules / cell | similar to [40] |
| *RNAPolII* | 5.8⋅10^3^ | molecules / cell | avg. value of several^#^ RNA Pol II subunits [16] |

*: stationary population average, use either 0 or 1 as initial condition

#: Rpb2, Rpb3, Rpb7, Rpb8, Rpb9, Rpb10, Rpb11, Rgr1, Rpc10, and Rpo26

### Simulation Settings

# For simulating the stochastic models, we used the approximative R-leaping algorithm by Auger *et al* [41], using their leaping condition 3 with the parameter settings *ε* = 0.05 (allowed fractional change in the propensity sum), *θ* = 0.5 (controlling the probability of negative species occurrence), and a reordering of the propensity vector at every *p_s_* = 100 time steps. These settings allowed to greatly speed up simulations (by more than a factor of 20 for our system compared to the Stochastic Simulation Algorithm due to Gillespie [42]), with negligible loss of accuracy. A C-based implementation of the algorithm was kindly provided and adapted for cluster computation by the Koumoutsakos group.

### Parameter Variation Studies

In the parameter studies addressing RPmRNA noise for the promoter designs 1 and 4, the gene inactivation rate constant was kept at its nominal value (see Table S6.Table S6) while varying the probability of an RP gene being active in the absence of TF binding by adjusting *K_a_* according to equation .

To decouple adjustment of protein and noise levels when varying Ifh1 properties, we employed the protein noise approximation suggested by Paulsson [43]:

$$\overbrace{CV_{TF_{i}}^{2}=\frac{\sigma_{TF_{i}}^{2}}{\left\langle n_{TF_{i}}\right\rangle^{2}}}^{\begin{matrix} \text{Total} \\ \text{protein} \\ \text{noise} \end{matrix}}=\overbrace{\underbrace{\frac{1}{\left\langle n_{TF_{i}}\right\rangle}}_{\text{Poisson}}}^{\begin{matrix} \text{from individual} \\ \text{births and deaths} \end{matrix}}+\overbrace{\underbrace{\frac{1}{\left\langle n_{TF_{i}mRNA}\right\rangle}}_{\text{Poisson}}\frac{\tau_{TF_{i}mRNA}}{\tau_{TF_{i}mRNA}+\tau_{TF_{i}}}}^{\text{from spontaneous mRNA noise}}$$

The symbol *σ_k_* stands for the standard deviation, *τ_k_* denotes the half life, and $\left\langle n_{k}\right\rangle$ represents the stationary ensemble average of species *k*. This equation assumes a constantly active promoter, which is the case for our model of TF synthesis, but neglects the effect of TF binding to target gene promoters. When varying *CVIfh1_T*, we modified *k_D,Ifh1_* and adjusted *k_TL,Ifh1_* accordingly to ensure a constant mean level of total Ifh1, which deviated less than 3 % from the average for all parameter combinations tested. By contrast, when varying Ifh1 levels, the expected contribution of spontaneous protein noise to total noise based on experimental data [16,35,36] is small (< 3 % of total noise acc. to equation ) even for the lowest Ifh1 level considered (72 molecules). Therefore, we maintained total protein noise at $CV_{Ifh1\_T}=50\%_{-10\%\min}^{+4\%\max}$ solely by adjusting *k_TL,Ifh1_* while leaving *τ_Ifh1_*, and hence *k_D,Ifh1_*, at its nominal value. In this way, *CVIfh1_T* was higher in Model 4 than in Model 1 by more than 6 % (but no more than 10 %) only for four settings (with *K_a_* ≤ 0.025 and *Ifh1_T_* ≤ 207 molecules) out of the total 121 settings tested and usually much less. However, the simulated difference in mRNA noise between Model 4 and Model 1 for these four particular settings was so large (Figure 5 E and F) that it could not be solely explained by the associated difference in *CVIfh1_T* levels. Indeed, nearly identical noise differences were observed for *K_a_* ≤ 0.025 and *Ifh1_T_* = 343 molecules (Figure 5 E and F) where *CV_Ifh1T_* differed by only 2 % between both models. This underlines that the observed higher noise of mRNA levels in Model 4 are indeed due to different model properties and do not represent an artifact introduced by small differences in *CVIfh1_T*  for equal parameter settings in the course of simulations.

### Quantification of Coregulation

We quantified the degree of mRNA coexpression from individual but identical RP gene promoters using the sum of squared pairwise differences between mRNA molecule numbers in a single run over time. For this analysis, we selected trajectory parts (75 time points) corresponding to stationary stochastic fluctuations, calculated the sum, and repeated the procedure for 500 individual simulation runs. A lower value of the sum indicates a higher degree of transcriptional coexpression. We, therefore, compared the mean values of the resulting distributions for Model 1 and Model 4, which were markedly different (2.20⋅10^4^ vs. 8.55⋅10^4^ molecules^2^). For sufficiently large degrees of freedom (here: the number of time points used to calculate each sum), the distribution of the sum of squares tends towards a normal distribution according to the central limit theorem [44]. This enabled us to assess the statistical significance of the difference in mean values using Welch’s t‑test for distributions with unequal variance, resulting in a highly significant outcome (p < 10^‑36^).

### Model Validation

*Galactose-induced IFH1 expression*

For galactose-induced *IFH1* expression (Figure 7), promoter occupancies and relative mRNA levels were taken from Wade *et al*. ([12], their Figure 3) and Schawalder *et al*. ([13], their Figure 3) and rescaled to yield fold-changes relative to the initial values of the individual RP genes where needed. Basal *IFH1* expression on the non-inducing carbon source was represented in the model by adjusting the synthesis rate of *IFH1*mRNA and simulating into (stochastic) steady state. We mimicked galactose induction at *t* = 0 by increasing *IFH1*mRNA synthesis to its induced rate after a lag time of 2 min, after which first changes in *GAL1*mRNA levels are observable experimentally [45]. All other model parameters remained at the nominal WT settings and *n* = 10,000 or 50,000 simulations were performed to determine average trajectories.

Since absolute values of *IFH1* mRNA expression on neutral carbon sources were not determined in both experimental datasets used, we varied the basal expression level in a plausible range (0.75-15% of the level on glucose) and determined the corresponding synthesis rates of *IFH1* mRNA by fitting to the measured time profile of relative *IFH1* mRNA abundance [13] using the deterministic model and the Evolutionary Programming routine of COPASI [46]. For this parameter estimation, separate values of the rate constant were determined for the increasing and decreasing portion of the time course considering simple step changes in the constant at the 2 min and 23 min time points (where the end point was treated as an additional parameter for optimization). The estimated parameters were then used to perform stochastic simulations as shown in Figure 7 B and C.

*Stress response*

Stress‑induced changes in total Fhl1 and Ifh1 protein levels were estimated by quantifying the Western blot data of Wade et al. ([12], their Figure 4 e) with ImageJ. Apart from these levels, all other model parameters were unaltered. We used data for the genes RPL2B, RPL27, and RPS11B for which both promoter occupancy and RPmRNA levels had been measured. Predicted promoter activities and occupancies by Rap1 and Fhl1 were computed with the deterministic model for varying effective Ifh1 concentrations. We used the measured Ifh1 occupancy of the respective RP genes (Figure 4d in [12]) to determine the corresponding values of activity and Rap1 and Fhl1 occupancy. Since the predicted stationary mRNA levels are proportional to promoter activity, we determined relative mRNA changes by the ratio of simulated promoter activities for the average measured Ifh1 occupations of promoters in a given stress condition. Predicted activities were related to promoter activity under nominal conditions (no stress) to yield the predicted mRNA ratio. We conducted simulations with basal transcriptional efficiencies of *η* = 0.05 and *η* = 0.2 for the *Rap1_2_RPGene* complex. Error bars for measured / predicted mRNA data indicate the standard deviation of the mean averaged over measured / predicted mean values for individual RP genes. For predicted promoter occupancies, error bars denote the standard deviation introduced by accounting for the mean standard deviation of Ifh1 occupancy averaged over individual RP genes. We assessed statistical significance of the difference between measured and simulated mean values using Welch’s *t*‑ test (two-tailed, confidence level 95%).

### SBML Models

A set of SBML models are included in the supplementary material, which contain the extended models 1 and 4 described above. For both models, a deterministic version (*Yeast_RP_Promoter_extd_Model1_det.xml* and *Yeast_RP_Promoter_extd_Model4_det.xml*) and a stochastic version (*Yeast_RP_Promoter_extd_Model1_stoch.xml* and *Yeast_RP_Promoter_extd_Model4_stoch.xml*) are included as Protocols S2 – S5. The model files are compliant with SBML Level 2 Version 1 and have been tested using JDesigner of the Systems Biology Workbench (v. 2.7.4, available from [http://www.sys-bio.org](http://www.sys-bio.org/)) with the Jarnac Simulation Service for deterministic models or in combination with the Dizzy Simulation Package (v. 1.11.4, available from <http://magnet.systemsbiology.net/software/Dizzy>) for stochastic models.

**References**

1. Wijchers P, Burbach JPH, Smidt MP (2006) In control of biology: of mice, men and Foxes. Biochemical Journal 397: 233-246.

2. Cherel I, Thuriaux P (1995) The Ifh1 Gene-Product Interacts with a Fork Head Protein in *Saccharomyces cerevisiae*. Yeast 11: 261-270.

3. Wittenberg C, Reed SI (2005) Cell cycle-dependent transcription in yeast: promoters, transcription factors, and transcriptomes. Oncogene 24: 2746-2755.

4. Ho Y, Gruhler A, Heilbut A, Bader GD, Moore L, et al. (2002) Systematic identification of protein complexes in *Saccharomyces cerevisiae* by mass spectrometry. Nature 415: 180-183.

5. Martin DE, Soulard A, Hall MN (2004) TOR regulates ribosomal protein gene expression via PKA and the forkhead transcription factor FHL1. Cell 119: 969-979.

6. Lieb JD, Liu XL, Botstein D, Brown PO (2001) Promoter-specific binding of Rap1 revealed by genome-wide maps of protein-DNA association. Nature Genetics 28: 327-334.

7. Morse RH (2000) RAP, RAP, open up! New wrinkles for RAP1 in yeast. Trends in Genetics 16: 51-53.

8. Boros J, Lim FL, Darieva Z, Pic-Taylor A, Harman R, et al. (2003) Molecular determinants of the cell-cycle regulated Mcm1p-Fkh2p transcription factor complex. Nucleic Acids Research 31: 2279-2288.

9. Freddie CT, Ji Z, Marais A, Sharrocks AD (2007) Functional interactions between the Forkhead transcription factor FOXK1 and the MADS-box protein SRF. Nucleic Acids Research 35: 5203-5212.

10. Buchwalter G, Gross C, Wasylyk B (2004) Ets ternary complex transcription factors. Gene 324: 1-14.

11. Sharrocks AD (2002) Complexities in ETS-Domain Transcription Factor Function and Regulation: Lessons from the TCF (Ternary Complex Factor) Subfamily. Biochemical Society Transactions 30: 1-9.

12. Wade JT, Hall DB, Struhl K (2004) The transcription factor Ifh1 is a key regulator of yeast ribosomal protein genes. Nature 432: 1054-1058.

13. Schawalder SB, Kabani M, Howald I, Choudhury U, Werner M, et al. (2004) Growth-regulated recruitment of the essential yeast ribosomal protein gene activator Ifh1. Nature 432: 1058-1061.

14. Harbison CT, Gordon DB, Lee TI, Rinaldi NJ, Macisaac KD, et al. (2004) Transcriptional regulatory code of a eukaryotic genome. Nature 431: 99-104.

15. Hall DB, Wade JT, Struhl K (2006) An HMG protein, Hmo1, associates with promoters of many ribosomal protein genes and throughout the rRNA gene locus in *Saccharomyces cerevisiae*. Molecular and Cellular Biology 26: 3672-3679.

16. Ghaemmaghami S, Huh W, Bower K, Howson RW, Belle A, et al. (2003) Global analysis of protein expression in yeast. Nature 425: 737-741.

17. Rudra D, Zhao Y, Warner JR (2005) Central role of Ifh1p-Fhl1p interaction in the synthesis of yeast ribosomal proteins. The EMBO Journal 24: 533-542.

18. Jorgensen P, Rupes I, Sharom JR, Schneper L, Broach JR, et al. (2004) A dynamic transcriptional network communicates growth potential to ribosome synthesis and critical cell size. Genes & Development 18: 2491-2505.

19. Zhao Y, McIntosh KB, Rudra D, Schawalder S, Shore D, et al. (2006) Fine-structure analysis of ribosomal protein gene transcription. Molecular and Cellular Biology 26: 4853-4862.

20. Cipollina C, van den Brink J, Daran-Lapujade P, Pronk JT, Vai M, et al. (2008) Revisiting the role of yeast Sfp1 in ribosome biogenesis and cell size control: a chemostat study. Microbiology 154: 337-346.

21. Horn F, Jackson R (1972) General Mass Action Kinetics. Archive for Rational Mechanics and Analysis 47: 81-&.

22. Feinberg M (1972) Chemical-Kinetics of a Certain Class. Archive for Rational Mechanics and Analysis 46: 1-&.

23. Walker GM (1998) Yeast - Physiology and Biotechnology. Chichester: John Wiley & Sons. 350 p.

24. Sherman F (2002) Getting started with yeast. In: Guthrie C, Fink GR, editors. Guide to Yeast Genetics and Molecular and Cell Biology, Pt B. San Diego: Academic Press. pp. 3-41.

25. Fraser HB, Hirsh AE, Giaever G, Kumm J, Eisen MB (2004) Noise minimization in eukaryotic gene expression. PLoS Biology 2: 834-838.

26. Blake WJ, Kaern M, Cantor CR, Collins JJ (2003) Noise in eukaryotic gene expression. Nature 422: 633-637.

27. Vignais ML, Woudt LP, Wassenaar GM, Mager WH, Sentenac A, et al. (1987) Specific Binding of Tuf Factor to Upstream Activation Sites of Yeast Ribosomal-Protein Genes. The EMBO Journal 6: 1451-1457.

28. Buck MJ, Lieb JD (2006) A chromatin-mediated mechanism for specification of conditional transcription factor targets. Nature Genetics 38: 1446-1451.

29. Rohde JR, Cardenas ME (2003) The tor pathway regulates gene expression by linking nutrient sensing to histone acetylation. Molecular and Cellular Biology 23: 629-635.

30. Geng FQ, Laurent BC (2004) Roles of SWI/SNF and HATs throughout the dynamic transcription of a yeast glucose-repressible gene. The EMBO Journal 23: 127-137.

31. Kuepfer L, Peter M, Sauer U, Stelling J (2007) Ensemble modeling for analysis of cell signaling dynamics. Nature Biotechnology 25: 1001-1006.

32. Elf J, Li GW, Xie XS (2007) Probing transcription factor dynamics at the single-molecule level in a living cell. Science 316: 1191-1194.

33. Buchler NE, Gerland U, Hwa T (2003) On schemes of combinatorial transcription logic. Proceedings of the National Academy of Sciences of the United States of America 100: 5136-5141.

34. Ullah M, Schmidt H, Cho KH, Wolkenhauer O (2006) Deterministic modelling and stochastic simulation of biochemical pathways using MATLAB. IEE Proceedings Systems Biology 153: 53-60.

35. Holstege FCP, Jennings EG, Wyrick JJ, Lee TI, Hengartner CJ, et al. (1998) Dissecting the regulatory circuitry of a eukaryotic genome. Cell 95: 717-728.

36. Belle A, Tanay A, Bitincka L, Shamir R, O'Shea EK (2006) Quantification of protein half-lives in the budding yeast proteome. Proceedings of the National Academy of Sciences of the United States of America 103: 13004-13009.

37. Mason PB, Struhl K (2005) Distinction and relationship between elongation rate and processivity of RNA polymerase II in vivo. Molecular Cell 17: 831-840.

38. Hurowitz EH, Brown PO (2003) Genome-wide analysis of mRNA lengths in *Saccharomyces cerevisiae*. Genome Biology 5: R2.

39. SGD-Project Saccharomyces Genome Database.

40. Warner JR (1999) The economics of ribosome biosynthesis in yeast. Trends in Biochemical Sciences 24: 437-440.

41. Auger A, Chatelain P, Koumoutsakos P (2006) R-leaping: Accelerating the stochastic simulation algorithm by reaction leaps. Journal of Chemical Physics 125: 084103.

42. Gillespie DT (1977) Exact Stochastic Simulation of Coupled Chemical-Reactions. Journal of Physical Chemistry 81: 2340-2361.

43. Paulsson J (2005) Models of stochastic gene expression. Physics of Life Reviews 2: 157-175.

44. Wasserman LA (2004) All of Statistics: A Concise Course in Statistical Inference. Berlin: Springer. 442 p.

45. Bryant GO, Ptashne M (2003) Independent Recruitment In Vivo by Gal4 of Two Complexes Required for Transcription. Molecular Cell 11: 1301-1309.

46. Hoops S, Sahle S, Gauges R, Lee C, Pahle J, et al. (2006) COPASI--a COmplex PAthway SImulator. Bioinformatics 22: 3067-3074.
